# ENPP1 deficiency: A clinical update on the relevance of individual variants using a locus‐specific patient database

**DOI:** 10.1002/humu.24477

**Published:** 2022-10-08

**Authors:** Stephanie A. Mercurio, Lauren M. Chunn, Gus Khursigara, Catherine Nester, Kathleen Wray, Ulrike Botschen, Mark J. Kiel, Frank Rutsch, Carlos R. Ferreira

**Affiliations:** ^1^ Department of Data Science, Curation Division Genomenon Inc. Ann Arbor Michigan USA; ^2^ Department of Scientific Communication and Strategy Genomenon Inc. Ann Arbor Michigan USA; ^3^ Department of Medical Affairs Inozyme Pharma Boston Massachusetts USA; ^4^ Department of Physician and Patient Strategies Inozyme Pharma Boston Massachusetts USA; ^5^ Department of General Paediatrics Muenster University Children's Hospital Münster Germany; ^6^ Metabolic Medicine Branch, National Human Genome Research Institute National Institutes of Health Bethesda Maryland USA

**Keywords:** autosomal recessive hypophosphatemic rickets type 2 (ARHR2), bone/Joint abnormalities, database, ENPP1 deficiency, generalized arterial calcification of infancy (GACI), genotype‐phenotype correlations, monoallelic ENPP1, rare variants

## Abstract

Loss‐of‐function variants in the ectonucleotide pyrophosphatase/phosphodiesterase family member 1 (ENPP1) cause ENPP1 Deficiency, a rare disorder characterized by pathological calcification, neointimal proliferation, and impaired bone mineralization. The consequence of ENPP1 Deficiency is a broad range of age dependent symptoms and morbidities including cardiovascular complications and 50% mortality in infants, autosomal recessive hypophosphatemic rickets type 2 (ARHR2) in children, and joint pain, osteomalacia and enthesopathies in adults. Recent research continues to add to the growing clinical presentation profile as well as expanding the role of ENPP1 itself. Here we review the current knowledge on the spectrum of clinical and genetic findings of ENPP1 Deficiency reported in patients diagnosed with GACI or ARHR2 phenotypes using a comprehensive database of known *ENPP1* variants with associated clinical data. A total of 108 genotypes were identified from 154 patients. Of the 109 *ENPP1* variants reviewed, 72.5% were demonstrably disease‐causing, a threefold increase in pathogenic/likely pathogenic variants over other databases. There is substantial heterogeneity in disease severity, even among patients with the same variant. The approach to creating a continuously curated database of *ENPP1* variants accessible to clinicians is necessary to increase the diagnostic yield of clinical genetic testing and accelerate diagnosis of ENPP1 Deficiency.

## 1 INTRODUCTION

The expanding role of ectonucleotide pyrophosphatase/phosphodiesterase family member 1 (ENPP1) in the pathogenesis of ectopic mineralization and paradoxical impairment of bone mineralization continues to garner interest. Loss‐of‐function variants in the *ENPP1* gene cause ENPP1 Deficiency, a rare disorder characterized by low pyrophosphate (inorganic pyrophosphate [PPi]) levels, excessive soft tissue calcification, arterial stenoses, and hypomineralization of bone (Ferreira, Hackbarth, et al., 2021; Nitschke et al., 2018; Rutsch et al., 2008). Early mortality, cardiac complications, hearing loss, impaired bone mineralization and ligament calcification are the clinical consequences of ENPP1 Deficiency resulting in significant morbidity in these patients (Ferreira, Ansh, et al., 2022; Ferreira, Hackbarth, et al., 2021; Ferreira, Kintzinger, et al., 2021; Saito et al., 2011; Theng et al., 2022).

Current research in ENPP1 continues to elucidate the heterogeneity of clinical presentation and morbidity across the age spectrum. ENPP1 Deficiency was initially well documented in patients presenting with generalized arterial calcification of infancy (GACI) (Rutsch et al., 2008). Neonates and infants present with extensive ectopic calcification, cardiovascular (CV) complications and a high mortality rate at infancy (Ferreira, Kintzinger, et al., 2021; Rutsch et al., 2008). These infants often first present with severe on‐specific CV symptoms such as hypertension and heart failure, for which numerous etiologies exist are often the first presentation (Ferreira, Kintzinger, et al., 2021; Rutsch et al., 2008). Adults and children who survive the early phase of the disease or do not present at the early phase infant stage of the disease may go on to develop hypophosphatemic rickets often reported as autosomal recessive hypophosphatemic rickets type 2 (ARHR2) and hearing loss (Ferreira, Hackbarth, et al., 2021; Ferreira, Kintzinger, et al., 2021; Theng et al., 2022). The clinical and biochemical characteristics of ARHR2 closely resemble X‐linked hypophosphatemia (XLH) and other genetic forms of rickets, including short stature, bone deformities and pain, gait abnormalities, renal phosphate wasting, and elevated FGF23 (Haffner et al., 2019; Levy‐Litan et al., 2010). Adults can go on to present with nonspecific symptoms such as joint pain, osteomalacia, and enthesopathies due to ENPP1 Deficiency (Ferreira, Ansh, et al., 2022; Oheim et al., 2020). Variants in the *ENPP1* gene have been identified in adult patients presenting with ossification of the posterior longitudinal ligament (OPLL) (Ferreira, Ansh, et al., 2022; Nakamura et al., 1999; Saito et al., 2011). While ENPP1 Deficiency is defined as an autosomal recessive disorder, there is growing evidence from case reports of adults with monoallelic *ENPP1* variants who presented with early‐onset osteoporosis and fractures (H. Kato et al., 2022; Oheim et al., 2020).

Due to the heterogeneity and nonspecific symptoms associated with ENPP1 Deficiency, an accurate diagnosis relies on genetic confirmation (Ferreira, Ansh, et al., 2022; Ferreira, Hackbarth, et al., 2021; Ferreira, Kintzinger, et al., 2021). However, the small number of patients diagnosed with this ultra‐rare disease (genetic prevalence estimated as 1/200,000 pregnancies) (Ferreira, Hackbarth, et al., 2021), coupled with the relatively high frequency of private variants—found in single families or a small number of individuals—pose a challenge to variant interpretation by clinicians and geneticists. To flatten the variants of uncertain significance (VUS) curve, it is critical to capture all clinical and functional evidence available to support variant reclassification. The aim of this review is to summarize current knowledge on the spectrum of clinical with genetic findings in patients with ENPP1 Deficiency. Specifically, this analysis included patients with ENPP1 variant(s) and a diagnosis of GACI or ARHR2 as this currently represents the most common phenotypes in the literature, with particular interest in pathogenic variants shaping the clinical presentation.

## 2 METHODOLOGY

### 2.1 Data sources and variant classification

We integrated data from a comprehensive, retrospective literature review with results from two natural history studies of GACI and ARHR2 patients to produce the most complete patient and variant database for ENPP1 Deficiency. Joining data from both sources is especially valuable for rare diseases given the difficulty in identifying and studying patients in numbers sufficient to be statistically meaningful. The variant database includes single nucleotide variants, small and large insertions/deletions (indels), as well as larger structural variants including exon‐level copy number variants and larger.

We identified and analyzed all published cases of ENPP1 Deficiency, specifically selecting for patients with a recorded diagnosis of GACI and/or ARHR2. The data was collated and all associated genetic variants in *ENPP1* were interpreted using a novel approach to systematic curation of genetic evidence. This comprehensive literature review was performed using the data content in Mastermind, a database of variants with evidence cited in the medical literature (Chunn et al., 2020) and considered all publications indexed from PubMed as of March 25th, 2021. A detailed description of the literature curation and bioinformatic processes used for variant interpretation are provided in the supplemental methods. Briefly, this technique combines automated indexing of medical literature with aggregation of population frequency databases and variant prediction algorithms followed by expert manual annotation and curation of this information and variant interpretation according to established clinical guidelines. In total, 2333 articles were reviewed.

In addition to published case reports, data was also collected from two recently published natural history studies, “Study of People with GACI or ARHR2” (identifier: NCT03478839) performed at the US National Institutes of Health (NIH), and “Natural History of GACI With or Without ARHR2 or PXE” (identifier: NCT03758534) performed in Germany's Münster University Children's Hospital (Ferreira, Kintzinger, et al., 2021). Data was also collected from two additional patients who were seen at the NIH but were not included in the natural history study.

Data from each source were exhaustively reviewed to ensure consistency in nomenclature and interpretation and to remove duplicate entries. When available from literature or from medical records, each patient was annotated with a detailed description of their phenotypic presentation and clinical outcome. Each variant was annotated with variant interpretations and detailed clinical and biochemical phenotypes extracted from literature reports and clinical testing submission forms. The nomenclature for each entry conforms to the Human Genome Variant Society guidelines (den Dunnen et al., 2016). Variant interpretations based on the consensus guidelines from the American College of Medical Genetics and Genomics and the Association for Molecular Pathology are included, as reported by a Clinical Laboratory Improvement Amendments certified clinical testing laboratory (“ACMG variant call”) or as predicted using variant interpretation software (“ACMG variant predictions”) (Nykamp et al., 2017; Richards et al., 2015). The ACMG variant interpretations were based on manual review of curated evidence from the literature as well as data extracted from external databases for computational predictions (PolyPhen2 [Adzhubei et al., 2010] and SIFT [Sim et al., 2012]) and population frequency (gnomAD [Karczewski et al., 2020]).

### 2.2 Patient and variant database composition

The combined approach described 154 total patients—111 individual GACI and/or ARHR2 patients harboring one or more *ENPP1* variant were initially identified through the literature review (72.1%) to which an additional 41 patients (26.6%) were included from the two natural history studies, including 27 from the study conducted in Germany and 14 conducted at the NIH. Five patients, two of which were not previously published (1.3%), were seen at the NIH but were not included in either of the two natural history studies. Of note, 45 out of the 111 patients (40.5%) initially identified in the literature analysis were catalogued in either or both natural history studies (including 17 identified in the German study, 26 identified in the NIH study and 2 identified in both studies).

An additional 4 patients with monoallelic ENPP1 Deficiency were identified, but were excluded from the analysis on the basis that they did not have a recorded diagnosis of GACI or ARHR2. Three of these patients were instead diagnosed with early‐onset osteoporosis, and one patient was diagnosed with pseudoxanthoma elasticum.

**Figure 1:**
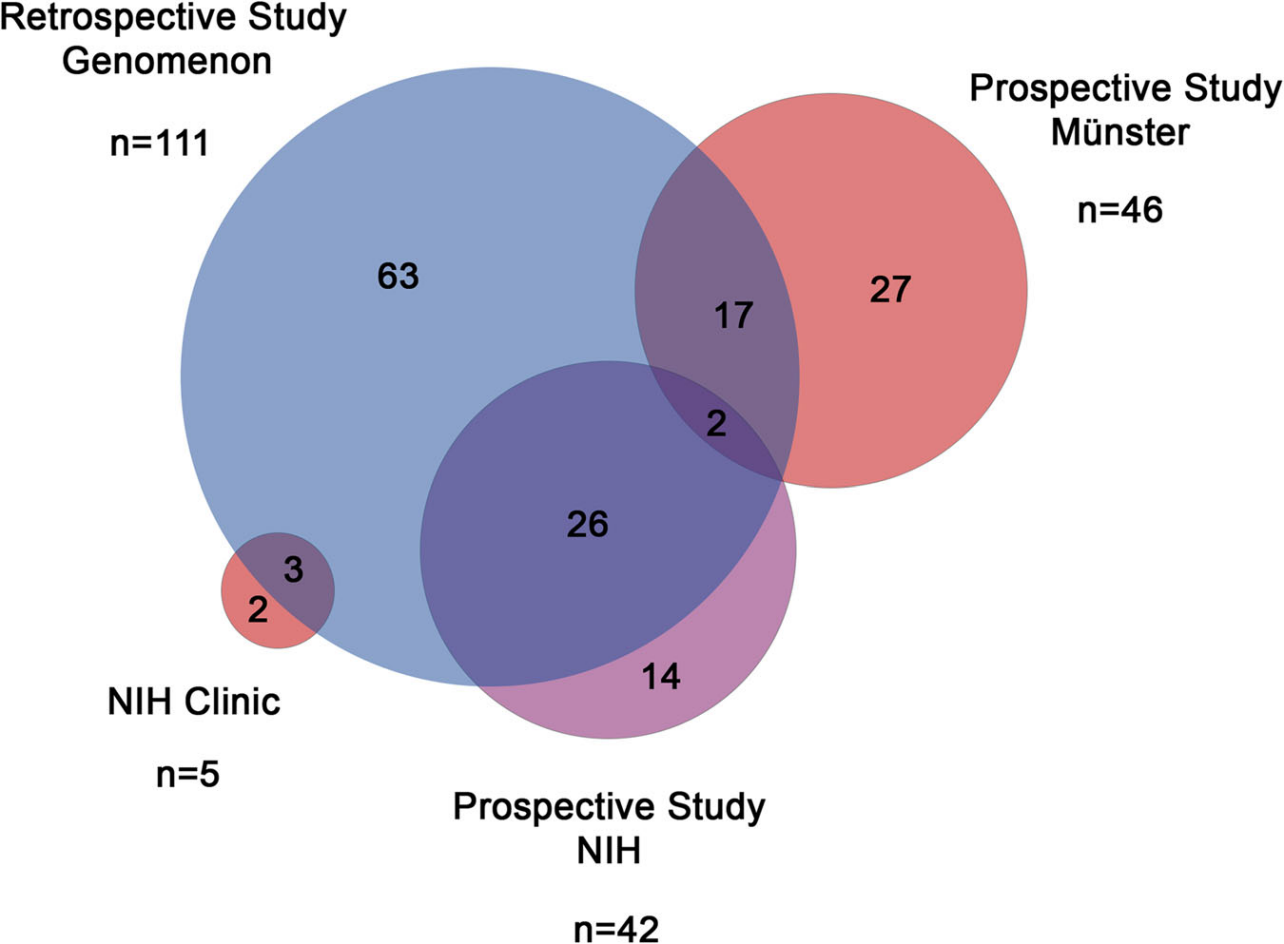
Source of GACI/ARHR2 patient information for the present study. Retrospective analysis of the published literature was performed using the Genomenon database of genetic evidence. Two distinct prospective natural history studies of GACI/ARHR2 patients were independently performed at University Children's Hospital Münster and at the National Institute of Health (NIH). The overlap of the origin of each patient is depicted. Care was taken to ensure no duplication of patient records occurred. ARHR2, autosomal recessive hypophosphatemic rickets type 2; GACI, generalized arterial calcification of infancy

In our literature review, extensive effort was expended to ensure patients were not double counted so it is presumed that the unpublished patients identified in the natural history studies would not have otherwise been encountered. A summary of the origin of each patient is depicted in Figure 1 underscoring the benefit of taking a combined approach to patient identification and characterization to maximize understanding of this rare disease. The combined results of these two approaches are presented in Table 1 including 108 unique genotypes and a high‐level summary of the clinical diagnostic data for each patient identified in both the retrospective and the natural history studies.

**Table 1:** *ENPP1* genotypes identified in the retrospective and prospective analyses of GACI/ARHR2 patients

Patient ID	Allele 1		Allele 2					
	cDNA (NM_006208.3)	Protein (NP_006199.2)	cDNA (NM_006208.3)	Protein (NP_006199.2)	Diagnosis	Age of Onset	Patient Source	PMID
1	c.[1831C>G;2002G>A]	p.[Leu611Val;Glu668Lys]	c.2375A>G	p.Asn792Ser	GACI	Neonatal	Literature | NIH NHS | Münster NHS	19229237 | 20016754 | 27467858
2	c.[1831C>G;2002G>A]	p.[Leu611Val;Glu668Lys]	c.2375A>G	p.Asn792Ser	GACI	Neonatal	Literature | NIH NHS | Münster NHS	19229237 | 20016754 | 12881724 | 27467858
3	c.913C>A	p.Pro305Thr	c.913C>A	p.Pro305Thr	GACI	Neonatal	Literature only	27467858
4	c.913C>A	p.Pro305Thr	c.913C>A	p.Pro305Thr	GACI	Antenatal	Literature only	27467858 | 20016754
5	c.913C>A	p.Pro305Thr	c.913C>A	p.Pro305Thr	GACI	Neonatal	Literature only	27467858 | 20016754
6	c.913C>A	p.Pro305Thr	c.913C>A	p.Pro305Thr	GACI	Neonatal	Literature only	27467858 | 20016754
7	c.913C>A	p.Pro305Thr	c.913C>A	p.Pro305Thr	GACI	Neonatal	Literature only	27467858 | 20016754 | 15605415
8	c.913C>A	p.Pro305Thr	c.913C>A	p.Pro305Thr	GACI	Antenatal	Literature only	27467858 | 20016754 | 1447660
9	c.913C>A	p.Pro305Thr	c.749C>T	p.Pro250Leu	GACI	Neonatal	Literature | NIH NHS	27467858 | 20016754 | 15605415 | 29244957
10	c.913C>A	p.Pro305Thr	c.749C>T	p.Pro250Leu	GACI | HR | PXE	Neonatal	Literature | NIH NHS	27467858 | 20016754 | 15605415 | 29244957 | 33005041 | 33465815
11	c.913C>A	p.Pro305Thr	c.1426C>T	p.Arg476*	GACI	Unknown	Literature only	27467858 | 20016754
12	c.913C>A	p.Pro305Thr	c.1499A>C	p.His500Pro	GACI	Unknown	Literature | NIH NHS	27467858 | 20016754 | 29244957
13	c.913C>A	p.Pro305Thr	c.557‐2A>G	p.?	GACI	Unknown	Literature only	27467858 | 20016754
14	c.913C>A	p.Pro305Thr	c.1164+2T>A	p.?	GACI | ARHR2	Neonatal	Literature | NIH NHS	27467858 | 20016754 | 16315058 | 15605415
15	c.913C>A	p.Pro305Thr	c.1164+2T>A	p.?	GACI | HR	Unknown	Literature | NIH NHS	27467858 | 20016754 | 16315058 | 15605415
16	c.[2320C>T;2662C>T]	p.[Arg774Cys;Arg888Trp]	c.913C>A	p.Pro305Thr	GACI	Neonatal	Literature | Münster NHS	27467858 | 20016754 | 15605415
17	c.913C>A	p.Pro305Thr	c.1709A>G	p.Tyr570Cys	GACI	Neonatal	Literature | Münster NHS	27467858
18	c.913C>A	p.Pro305Thr	c.913C>A	p.Pro305Thr	GACI	Neonatal	Literature only	15605415
19	c.913C>A	p.Pro305Thr	c.1499A>C	p.His500Pro	GACI | ARHR2	Neonatal	Literature | NIH NHS	29244957 | 33005041 | 33465815
20	c.913C>A	p.Pro305Thr	c.2246C>G	p.Ser749*	GACI	Neonatal	Literature only	28973083
21	c.2320C>T	p.Arg774Cys	c.2320C>T	p.Arg774Cys	GACI | ARHR2	Antenatal	Literature | NIH NHS	27467858 | 20016754 | 12881724
22	c.[1737G>C;2320C>T]	p.[Leu579Phe;Arg774Cys]	c.1072_1082del	p.Gln358Argfs*2	GACI	Neonatal	Literature only	27467858 | 20016754 | 12881724
23	c.[2320C>T;2662C>T]	p.[Arg774Cys;Arg888Trp]	c.2375A>G	p.Asn792Ser	GACI	Unknown	Literature only	27467858 | 20016754
24	c.[2320C>T;2662C>T]	p.[Arg774Cys;Arg888Trp]	Not identified	Not identified	GACI	Unknown	Literature only	15605415
25	c.2320C>T	p.Arg774Cys	c.2320C>T	p.Arg774Cys	HR	Unknown	Literature only	20016754 | 12881724
26	c.2662C>T	p.Arg888Trp	c.2663G>A(;)2320C>T	p.Arg888Gln(;)Arg774Cys	ARHR2	Unknown	Literature only	33107440
27	c.2375A>G	p.Asn792Ser	c.1510A>C	p.Ser504Arg	GACI	Unknown	Literature only	20016754 | 27467858
28	c.2375A>G	p.Asn792Ser	c.936T>G	p.Tyr312*	GACI	Neonatal	Literature only	20016754 | 12881724
29	c.2662C>T	p.Arg888Trp	Not identified	Not identified	GACI	Unknown	Literature only	27467858
30	c.2662C>T	p.Arg888Trp	c.1709A>G	p.Tyr570Cys	GACI	Unknown	Literature only	27467858
31	c.797G>T	p.Gly266Val	c.797G>T	p.Gly266Val	ARHR2	Adult	Literature only	20137773
32	c.797G>T	p.Gly266Val	c.797G>T	p.Gly266Val	ARHR2	Juvenile	Literature only	20137773
33	c.797G>T	p.Gly266Val	c.797G>T	p.Gly266Val	GACI	Neonatal	Literature only	20137773
34	c.1112A>T	p.Tyr371Phe	c.1025G>T	p.Gly342Val	GACI	Neonatal	Literature only	15605415 | 15940697 | 20016754
35	c.1112A>T	p.Tyr371Phe	c.1025G>T	p.Gly342Val	GACI	Neonatal	Literature only	15605415 | 15940697 | 20016754
36	c.749C>T	p.Pro250Leu	c.783C>G	p.Tyr261*	ARHR2	Unknown	Literature only	26377240
37	c.1412A>G	p.Tyr471Cys	c.826G>A	p.Asp276Asn	GACI	Antenatal	Literature only	27467858 | 20016754
38	c.1412A>G	p.Tyr471Cys	c.826G>A	p.Asp276Asn	GACI	Neonatal	Literature | Münster NHS	27467858 | 20016754
39	c.1412A>G	p.Tyr471Cys	c.1426C>T	p.Arg476*	GACI	Neonatal	Literature only	27467858
40	c.1412A>G	p.Tyr471Cys	c.2311+1G>A	p.?	GACI	Unknown	Literature only	27467858
41	c.1412A>G	p.Tyr471Cys	c.1709A>G	p.Tyr570Cys	GACI	Neonatal	Literature only	27467858 | 20016754
44	c.1412A>G	p.Tyr471Cys	c.1442G>A	p.Arg481Gln	GACI | ARHR2	Infantile	Literature | NIH NHS	29244957 | 33005041 | 26857895 | 33465815
45	c.1538A>G	p.Tyr513Cys	c.1976A>G	p.Tyr659Cys	GACI	Neonatal	Literature only	27467858 | 20016754
46	c.1538A>G	p.Tyr513Cys	c.1538A>G	p.Tyr513Cys	PXE | GACI	Neonatal	Literature | NIH NHS	22229486 | 29244957 | 33005041 | 33465815
47	c.[2320C>T;2662C>T]	p.[Arg774Cys;p.Arg888Trp]	c.913C>A	p.Pro305Thr	GACI	Neonatal	Literature | Münster NHS	15605415
48	c.2311+1G>A	p.?	Not identified	Not identified	HR	Childhood	Literature only	26051471
49	c.323G>T	p.Cys108Phe	c.1441C>T	p.Arg481Trp	ARHR2	Adult	Literature | NIH NHS	31826312
50	c.323G>T	p.Cys108Phe	c.1441C>T	p.Arg481Trp	ARHR2	Neonatal	Literature only	31826312
51	c.1441C>T	p.Arg481Trp	c.2713_2717del	p.Lys905Alafs*16	GACI	Unknown	Literature | NIH NHS	12881724 | 20016754
52	c.725G>A	p.Gly242Glu	c.1441C>T	p.Arg481Trp	GACI	Unknown	Literature only	20016754
53	c.783C>G	p.Tyr261*	c.878_879del	p.Lys293Argfs*4	GACI | PXE | CJ	Neonatal	Literature | Münster NHS	19206175 | 22209248 | 20016754 | 15605415
54	c.783C>G	p.Tyr261*	c.878_879del	p.Lys293Argfs*4	GACI	Antenatal	Literature only	19206175 | 20016754 | 15605415
56	c.1709A>G	p.Tyr570Cys	c.1046G>A	p.Arg349Lys	GACI	Neonatal	Literature only	20016754 | 16429273
57	c.1612G>C	p.Asp538His	c.1612G>C	p.Asp538His	PXE |GACI | HR	Neonatal	Literature | Münster NHS	27467858 | 22209248
59	c.376T>C	p.Cys126Arg	c.2176T>C	p.Cys726Arg	GACI	Neonatal	Literature | Münster NHS	20016754 | 12881724
60	c.2410G>C	p.Asp804His	c.[2410G>C;2462G>A]	p.[Asp804His;Arg821His]	GACI	Unknown	Literature only	20016754 | 27467858 | 15605415
61	c.2410G>C	p.Asp804His	c.[2410G>C;2462G>A]	p.[Asp804His;Arg821His]	GACI	Unknown	Literature only	20016754 | 27467858 | 15605415
62	c.2677G>T	p.Glu893*	c.2677G>T	p.Glu893*	GACI	Unknown	Literature | Münster NHS	20016754 | 12881724
63	c.647C>A	p.Ser216Tyr	c.647C>A	p.Ser216Tyr	GACI	Unknown	Literature only	20016754
64	c.753_755del	p.Tyr252del	c.753_755del	p.Tyr252del	GACI	Unknown	Literature | Münster NHS	20016754 | 15605415
65	c.[430+2T>C;2330A>G]	p.[?;His777Arg]	c.2330A>G	p.His777Arg	GACI	Unknown	Literature only	20016754 | 27467858
66	c.1367G>A	p.Arg456Gln	Not identified	Not identified	GACI	Unknown	Literature only	20016754 | 12881724
67	c.288del	p.Leu97*	c.2479_2482dup	p.Pro828Hisfs*13	GACI	Neonatal	Literature | Münster NHS	20016754
68	c.2713_2717del	p.Lys905Alafs*16	c.2713_2717del	p.Lys905Alafs*16	GACI	Unknown	Literature only	20016754
69	c.2248dup	p.Ser750Lysfs*6	c.2248dup	p.Ser750Lysfs*6	ARHR2	Juvenile	Literature only	20137773
70	c.583T>C	p.Cys195Arg	c.583T>C	p.Cys195Arg	GACI | ARHR2	Antenatal	Literature | Münster NHS	27467858 | 21932012
71	c.583T>C	p.Cys195Arg	c.583T>C	p.Cys195Arg	GACI	Unknown	Literature only	27467858
72	c.583T>C	p.Cys195Arg	Not identified	Not identified	GACI	Unknown	Literature only	27467858
73	c.583T>C	p.Cys195Arg	Not identified	Not identified	GACI	Unknown	Literature only	27467858
74	c.583T>A	p.Cys195Ser	c.902A>G	p.Tyr301Cys	GACI	Unknown	Literature only	27467858
75	c.1756G>A	p.Gly586Arg	c.795+1G>A	p.?	GACI | HR | PXE	Unknown	Literature | Münster NHS	27467858 | 22209248
76^a^	c.2462G>A	p.Arg821His	Not identified	Not identified	GACI	Unknown	Literature only	27467858
77	c.2212G>A	p.Gly738Arg	c.2212G>A	p.Gly738Arg	GACI	Antenatal	Literature only	28276217
155	c.2212G>A	p.Gly738Arg	c.2212G>A	p.Gly738Arg	GACI	Antenatal	Münster NHS	None
78	c.966G>A	p.Trp322*	c.966G>A	p.Trp322*	GACI	Neonatal	Literature only	28276217
80	c.755A>G	p.Tyr252Cys	c.755A>G	p.Tyr252Cys	ARHR2	Unknown	Literature only	31805212
81	c.755A>G	p.Tyr252Cys	c.755A>G	p.Tyr252Cys	ARHR2	Unknown	Literature only	31805212
83	c.556G>C	p.Gly186Arg	c.556G>C	p.Gly186Arg	GACI	Antenatal	Literature | NIH NHS	31444901
84	c.556G>C	p.Gly186Arg	c.556G>C	p.Gly186Arg	GACI	Antenatal	Literature | NIH NHS	31444901
85	c.556G>C	p.Gly186Arg	c.556G>C	p.Gly186Arg	GACI	Antenatal	Literature | NIH NHS	31444901
86	c.556G>C	p.Gly186Arg	c.556G>C	p.Gly186Arg	GACI	Neonatal	Literature | NIH NHS	31444901
87	c.2344C>T	p.Arg782*	c.2344C>T	p.Arg782*	GACI | HR	Antenatal	Literature only	16369898 | 19521093
88	c.2344C>T	p.Arg782*	c.2344C>T	p.Arg782*	GACI	Antenatal	Literature | Münster NHS	19521093
89	c.784A>G	p.Ser262Gly	c.784A>G	p.Ser262Gly	GACI	Neonatal	Literature only	29976176
90	c.2026C>T	p.Gln676*	c.[2375A>G;655G>A]	p.[Asn792Ser;Gly219Arg]	ARHR2	Childhood	Literature | Münster NHS	25741938
91	c.2230+1_2230+3delinsCACC	p.?	c.2230+1_2230+3delinsCACC	p.?	HR| OPLL	Adult	Literature only	21745613
92	c.2702A>C	p.Tyr901Ser	c.2702A>C	p.Tyr901Ser	ARHR2	Juvenile	Literature only	20137772
93	c.2702A>C	p.Tyr901Ser	c.2702A>C	p.Tyr901Ser	ARHR2	Juvenile	Literature only	20137772
94	c.2702A>C	p.Tyr901Ser	c.2702A>C	p.Tyr901Ser	ARHR2	Adult	Literature only	20137772
95	c.1438T>C	p.Cys480Arg	c.2414G>T	p.Gly805Val	GACI | LDS | ARHR2| PXE	Neonatal	Literature | NIH NHS	29244957 | 33005041 | 33465815
96	c.1438T>C	p.Cys480Arg	c.2414G>T	p.Gly805Val	GACI | ARHR2	Antenatal	Literature | NIH NHS	29244957 | 33005041 | 33465815
97	c.653A>T	p.Asp218Val	c.653A>T	p.Asp218Val	GACI	Antenatal	Literature only	23430823
156^a^	c.653A>T	p.Asp218Val	c.653A>T	p.Asp218Val	GACI	Unknown	Literature | Münster NHS	22972716
98	c.275G>A	p.Gly92Asp	c.2230+1G>A	p.?	ARHR2	Neonatal	Literature | Münster NHS	25741938
99	c.2444+702_*868del	p.?	c.2444+702_*868del	p.?	HR	Juvenile	Literature only	20137773
100	c.2444+702_*868del	p.?	c.2444+702_*868del	p.?	HR	Childhood	Literature only	20137773
101	c.1000C>G	p.Pro334Ala	c.1000C>G	p.Pro334Ala	GACI	Antenatal	Literature only	31742715
102	c.1441C>T	p.Arg481Trp	c.2312‐5_2313del	p.?	GACI | ARHR2	Infantile	Literature | NIH NHS	33005041 | 33465815
103	c.1441C>T	p.Arg481Trp	c.2312‐5_2313del	p.?	GACI | ARHR2	Childhood	Literature | NIH NHS	33005041 | 33465815
104	c.2735T>C	p.Leu912Ser	c.(617+1_618‐1)_(715+1_716‐1)del	p.?	GACI	Neonatal	Literature | NIH NHS	33005041 | 33465815
105	c.1441C>T	p.Arg481Trp	c.2713_2717del	p.Lys905Alafs*16	GACI | ARHR2	Antenatal	Literature | NIH NHS	33005041 | 33465815
106	c.749C>T	p.Pro250Leu	c.749C>T	p.Pro250Leu	GACI | ARHR2	Antenatal	Literature | NIH NHS	33005041 | 33465815
107	c.1652A>G	p.Tyr551Cys	c.2330A>G	p.His777Arg	GACI | BS2	Neonatal	Literature | NIH NHS	33005041
109	c.1652A>G	p.Tyr551Cys	c.1737G>C	p.Leu579Phe	GACI	Neonatal	Literature | NIH NHS	33005041 | 33465815
110	c.913C>A	p.Pro305Thr	c.2662C>T	p.Arg888Trp	GACI	Antenatal	Literature | NIH NHS	33005041 | 33465815
111	c.2320C>T	p.Arg774Cys	c.2662C>T	p.Arg888Trp	GACI | ARHR2 | PK	Antenatal	Literature | NIH NHS	33005041 | 33465815
112	c.803A>G	p.Tyr268Cys	c.2596G>A	p.Glu866Lys	GACI| HR| PXE	Childhood	Literature | NIH NHS	33005041
113	c.783C>G	p.Tyr261*	c.1756G>A	p.Gly586Arg	ARHR2	Unknown	Literature | NIH Clinic	33465815
114	c.715+1G>C	p.?	c.2376T>A	p.Asn792Lys	GACI | ARHR2	Unknown	Literature | NIH Clinic	33465815
115	c.715+1G>C	p.?	c.2376T>A	p.Asn792Lys	ARHR2	Unknown	Literature | NIH Clinic	33465815
116	c.1412A>G	p.Tyr471Cys	c.1715T>C	p.Leu572Ser	GACI	Antenatal	Literature only	34199854
117	c.956C>G	p.Thr319Arg	c.2344C>T	p.Arg782*	HR | OALL	Childhood	Literature only	22539483
58	c.196_197del	p.Ala66Profs*10	c.2230C>T	p.Gln744*	GACI | ARHR2	Unknown	Münster NHS	None
82	c.1441C>T	p.Arg481Trp	c.2664del	p.Ile889Serfs*24	GACI	Unknown	Münster NHS	None
108	c.208A>T	p.Lys70*	Not identified	Not identified	GACI	Unknown	Münster NHS	None
118^a^	c.1652A>G	p.Tyr551Cys	c.1652A>G	p.Tyr551Cys	GACI	Unknown	Münster NHS	None
119	c.1412A>G	p.Tyr471Cys	c.2311+1G>A	p.?	GACI	Unknown	Münster NHS	None
120	c.511A>T	p.Lys171*	c.574del	p.Glu192Lysfs*47	GACI | ARHR2	Infantile	Münster NHS	None
121	c.1367G>A	p.Arg456Gln	c.1367G>A	p.Arg456Gln	GACI	Unknown	Münster NHS	None
122	c.2320C>T	p.Arg774Cys	c.2230C>T	p.Gln744*	GACI	Neonatal	Münster NHS	None
123	c.1437+1G>T	p.?	Not identified	Not identified	GACI	Unknown	Münster NHS	None
124	c.1094del	p.Pro365Hisfs*8	c.1094del	p.Pro365Hisfs*8	GACI	Neonatal	Münster NHS	None
125	c.196_197del	p.Ala66Profs*10	c.[1026‐59_1026‐10del;−24G>C]	p.[?;?]	GACI	Neonatal	Münster NHS	None
126	c.26dup	p.Gly10Argfs*67	c.26dup	p.Gly10Argfs*67	GACI | ARHR2	Infantile	Münster NHS	None
128	c.130C>T	p.Gln44*	c.1112A>T	p.Tyr371Phe	GACI	Infantile	Münster NHS	None
129	c.749C>T	p.Pro250Leu	c.749C>T	p.Pro250Leu	GACI | ARHR2	Neonatal	Münster NHS	None
130	c.2192del	p.Asn731Ilefs*5	c.583T>C	p.Cys195Arg	GACI | ARHR2	Unknown	Münster NHS	None
131	c.1106C>T	p.Thr369Ile	c.2300del	p.Gln767Argfs*44	GACI	Neonatal	Münster NHS	None
132	c.2713_2717del	p.Lys905Alafs*16	c.2713_2717del	p.Lys905Alafs*16	GACI	Neonatal	Münster NHS	None
133	c.1652A>G	p.Tyr551Cys	c.1652A>G	p.Tyr551Cys	GACI	Neonatal	Münster NHS	None
135	c.2444+702_*868del	p.?	c.2444+702_*868del	p.?	ARHR2	Unknown	Münster NHS	None
136	c.797G>T	p.Gly266Val	Not identified	Not identified	ARHR2	Unknown	Münster NHS	None
137	c.583T>C	p.Cys195Arg	c.1412A>G	p.Tyr471Cys	ARHR2	Juvenile	Münster NHS	None
138	c.665C>A	p.Ala222Glu	c.665C>A	p.Ala222Glu	GACI	Neonatal	Münster NHS	None
139	c.749C>T	p.Pro250Leu	c.749C>T	p.Pro250Leu	GACI	Neonatal	NIH NHS	None
140	c.2663G>A	p.Arg888Gln	c.570G>T	p.Trp190Cys	GACI	Infantile	NIH NHS	None
141	c.(1091+1_1092‐1)_(1164+1_1165‐1)del	p.?	c.(1091+1_1092‐1)_(1164+1_1165‐1)del	p.?	GACI	Neonatal	NIH NHS	None
142	c.484A>G	p.Ser162Gly	Not identified	Not identified	GACI	Neonatal	NIH NHS	None
143	c.2741T>A	p.Leu914*	c.1412A>G	p.Tyr471Cys	GACI	Neonatal	NIH NHS	None
144	c.915+1G>A	p.?	c.1976A>G	p.Tyr659Cys	GACI | ARHR2	Neonatal	NIH NHS	None
145	c.913C>A	p.Pro305Thr	c.913C>A	p.Pro305Thr	GACI | ARHR2	Neonatal	NIH NHS	None
146	c.913C>A	p.Pro305Thr	c.913C>A	p.Pro305Thr	GACI	Neonatal	NIH NHS	None
147	c.913C>A	p.Pro305Thr	c.2663G>A	p.Arg888Gln	GACI	Neonatal	NIH NHS	None
148	c.913C>A	p.Pro305Thr	c.1273+2T>C	p.?	GACI	Neonatal	NIH NHS	None
149	c.1025G>T	p.Gly342Val	c.1068G>A	p.Trp356*	GACI	Infantile	NIH NHS	None
150	c.796G>A	p.Gly266Arg	c.−119399_*324497del	p.?	GACI	Neonatal	NIH NHS	None
151	c.1273+1G>A	p.?	c.1273+1G>A	p.?	ARHR2	Childhood	NIH NHS	None
152	c.241G>T	p.Val81Leu	c.241G>T	p.Val81Leu	ARHR2	Infantile	NIH NHS	None
153	c.1026‐281_1164+1delinsN[7]	p.?	c.1026‐281_1164+1delinsN[7]	p.?	GACI	Neonatal	Münster NHS	None
154	c.2713_2717del	p.Lys905Alafs*16	c.2713_2717del	p.Lys905Alafs*16	ARHR2	Neonatal	Münster NHS	None
157	c.1604A>G	p.His535Arg	c.2212G>A	p.Gly738Arg	GACI	Unknown	NIH Clinic	None
158	c.1437+1G>T	p.?	c.1652A>G	p.Tyr551Cys	ARHR2	Unknown	NIH Clinic	None
159	c.749C>T	p.Pro250Leu	c.749C>T	p.Pro250Leu	GACI	Antenatal	Münster NHS	None
160	c.1367G>A	p.Arg456Gln	c.1367G>A	p.Arg456Gln	GACI	Antenatal	Münster NHS	None

Abbreviations: ARHR2, autosomal recessive hypophosphatemic rickets type 2; BS2, Brugada syndrome type 2; CJ, Creutzfeldt‐Jakob; GACI, generalized arterial calcification of infancy; HR, hypophosphatemic rickets; LDS, Loeys‐Dietz syndrome; OALL, ossification of the anterior longitudinal ligament; OPLL, ossification of posterior longitudinal ligament; PK, Phenylketonuria; PXE Pseudoxanthoma elasticum.

^a^ Additional variant was found in patients #76 (ABCC6 NM_001171.5:c.3940C>T, p.Arg1314Trp), #156 (ABCC6 NM_001171.5:c.3340C>T, p.Arg1114Cys), and #118 (ABCC6 NM_001171.5:c.1540G>A, p.Val514Ile).

## 3 ENPP1 DEFICIENCY

### 3.1 ENPP1 protein structure and function

*ENPP1* is expressed in numerous tissues including osteoblasts and osteocytes, chondrocytes, vascular smooth muscle cells, renal proximal tubule epithelial cells, mature plasma cells, and skin fibroblasts (Goding et al., 2003). The *ENPP1* gene product is a type II transmembrane glycoprotein consisting of a small intracellular domain, a single transmembrane domain, and a larger extracellular domain containing a catalytic site (Goding et al., 2003). The cytoplasmic domain (residues 1–76) and transmembrane domain (residue 77‐97) localize the protein in the plasma membrane while two extracellular somatomedin B‐like domains (SMB1, residue 104–144) and SMB2, residues 145–188) are implicated in homodimerization (Bello et al., 2001; Vaingankar et al., 2004). The ENPP1 catalytic domain comprises a phosphodiesterase (residues 191–591) which is N‐terminally flanked by the SMB domains, and C‐terminally linked to a nuclease‐like domain (residues 654–925) (Jansen et al., 2012).

The ENPP1 catalytic domain cleaves the phosphodiester bonds of nucleotides, preferentially hydrolyzing extracellular ATP into PPi and adenosine monophosphate (AMP) (K. Kato et al., 2012; Zhou et al., 2012). PP_i_ acts as the main physiologic inhibitor of calcification by antagonizing hydroxyapatite formation and deposition (Fleisch & Bisaz, 1962; Goding et al., 2003; Zhou et al., 2012). AMP is further metabolized into adenosine, an inhibitor of neointimal proliferation (Albayrak et al., 2015; Dubey et al., 1998). Biallelic loss‐of‐function variants in the *ENPP1* gene are associated with low levels of PP_i_ and adenosine, leading to pathologic ectopic vascular calcification and neointimal proliferation, respectively (Nitschke et al., 2018).

### 3.2 Animal models of ENPP1 deficiency

Mouse models that recapitulate the clinical phenotypes of patients with ENPP1 Deficiency have been a valuable tool in understanding the disease mechanism and potential therapies targeting *ENPP1* mutations. The first mouse model of ENPP1 deficiency was the *Enpp1* ^*ttw/ttw*^ (“tiptoe walking”) mouse, harboring a homozygous p.Gly568Ter variant and characterized by ectopic mineralization of the spinal ligaments (Okawa et al., 1998). This mouse model has also demonstrated that ENPP1 functions to inhibit neointima formation by producing AMP, together linking the role of ENPP1 to regulating PP_i_ levels and arterial thickness (Nitschke et al., 2018). A mouse harboring a homozygous deletion of *Enpp1* had a phenotype essentially identical to that of the *Enpp1* ^*ttw/ttw*^ mouse (Johnson et al., 2003). *Enpp1* ^*asj/asj*^ (“ages with stiffened joint”) mice harboring the p.Val246Asp variant display low plasma PPi levels and vascular mineralization (Li et al., 2013). When fed a diet high in phosphate but low in magnesium, these *Enpp1* ^*asj/asj*^ mice showed accelerated mineralization and shorter lifespans. A fourth mouse model is the *Enpp1* ^*asj‐2J*^ mouse, harboring a large 40 kilobase deletion combined with a 74 bp insertion, and leading to more extensive mineralization than seen in the *Enpp1* ^*asj/asj*^ mice (Li et al., 2013). The *Enpp1* ^*asj/asj*^ and *Enpp1* ^*asj‐2J*^ models recapitulate the clinical features in humans with ENPP1 deficiency; low PPi, elevated FGF23, hypophosphatemia, arterial calcification, neointima proliferation, hearing loss, enthesopathies and early mortality as well as lower bone mass or osteomalacia, in heterozygous and homozygous mice (Cheng et al., 2021; Ferreira, Ansh, et al., 2022; Li et al., 2013, 2016; Oheim et al., 2020; Tian et al., 2016; Zimmerman et al., 2022). Introducing a recombinant ENPP1 protein in *Enpp1* ^*asj/asj*^ or *Enpp1* ^*asj‐2J*^ mice increases low PP_i_ levels, mitigates aberrant FGF23 elevation, prevents ectopic calcification and neointima proliferation, reduces mortality and prevents bone mass loss, elucidating the role of ENPP1 and suggesting exogenous replacement of ENPP1 as a possible therapeutic strategy possibly in homozygous and heterozygous ENPP1 deficient patients (Albright et al., 2015; Cheng et al., 2021; Ferreira, Ansh, et al., 2022; Ferreira, Kavanagh, et al., 2021; Maulding et al., 2021). A mutant zebrafish model also displays ectopic mineralization in soft tissues (Apschner et al., 2014).

A *Enpp1* ^*T238A*^ transgenic mouse model with a single point variant at amino acid 238, rendering the ENPP1 enzyme catalytically inactive while preserving its protein expression and signaling, was developed to distinguish between possible catalytic and noncatalytic functions of ENPP1 (Zimmerman et al., 2022). Similar to *Enpp1* ^*asj/asj*^ mice, *Enpp1* ^*T238A*^ mice demonstrate low plasma PPi, increased FGF23, ectopic calcifications, and reduced cortical thickness by week 23, suggesting ENPP1 catalytic activity impacts cortical bone mass. In contrast to *Enpp1* ^*asj/asj*^ mice, however, *Enpp1* ^*T238A*^ mice have normal trabecular bone mineral density and femur biomechanical strength, thus pointing to a role for catalysis‐independent ENPP1 protein signaling in the regulation of trabecular microarchitecture. Supporting this notion, Maulding et al. (2021) report a decrease in Wnt ligands Wnt10b and Wnt16 in *Enpp1* ^*asj/asj*^ and a similar skeletal phenotype between *Enpp1* ^*asj/asj*^ mice and Wnt10b‐ and Wnt16‐knockout mice, suggesting ENPP1 regulation of the Wnt signaling pathway, an important regulatory pathway in osteogenic differentiation of mesenchymal stem cells. ENPP1 transcript counts, compared to plasma PPi concentrations, correlated better to the skeletal phenotype of 10 weeks old in *Enpp1* ^*asj/asj*^ mice, suggesting a catalytically‐independent function of ENPP1. The authors continue to show this mechanism is through suppression of Wnt inhibitor secreted frizzled‐related protein 1 (SFRP1). Interestingly, this role shifts to the catalytic‐driven low PPi levels at 23 weeks.

### 3.3 ENPP1 deficiency clinical presentation and phenotypes

ENPP1 Deficiency is associated with significant morbidity and mortality across the age spectrum. There is considerable heterogeneity in the age of symptom onset, clinical presentation, and severity.

The clinical presentation of ENPP1 Deficiency in infants, typically described as GACI, is characterized by arterial calcification, stenosis of large and medium‐sized vessels and severe CV problems such as systemic hypertension, pulmonary hypertension, heart failure, cardiomyopathy or myocardial ischemia/infarction (Ferreira, Hackbarth, et al., 2021; Rutsch et al., 2008). Infants may also present with seizures or stroke, attributed to calcification of the cerebral arteries (Ferreira, Hackbarth, et al., 2021; Ferreira, Kintzinger, et al., 2021; Mulcahy et al., 2019). Calcification of organs and joints are also observed (Ferreira, Hackbarth, et al., 2021). Approximately 50% of infants with ENPP1 Deficiency die within the first 6 months of life despite receiving standard of care treatments (Ferreira, Kintzinger, et al., 2021; Rutsch et al., 2008).

Patients who survive infancy or who first exhibit symptoms of ENPP1 Deficiency in childhood typically present with FGF23‐mediated hypophosphatemic rickets, described as ARHR2 (Ferreira, Hackbarth, et al., 2021). This might represent a compensatory mechanism to mitigate vascular calcification, as increased FGF23 expression reduces renal phosphate reabsorption and promotes its excretion. In a prospective natural history study of patients with ENPP1 Deficiency a Kaplan–Meier analyses estimated that 90% of patients would develop hypophosphatemic rickets by age 15 (Ferreira, Hackbarth, et al., 2021). Additionally, a large portion of patients with ENPP1 Deficiency will also develop and present with hearing loss, joint calcification, or enthesopathies (Brachet et al., 2014; Ferreira, Ansh, et al., 2022; Kotwal et al., 2020; Theng et al., 2022; Thumbigere‐Math et al., 2018).

The CV and skeletal complications of ENPP1 Deficiency may continue into adulthood. Adult patients with ENPP1 Deficiency often present with symptoms of osteomalacia or late‐onset musculoskeletal complications, including bone and joint pain and enthesopathies impacting daily function (Ferreira, Ansh, et al., 2021; Ferreira, Hackbarth, et al., 2021). CV or renal disease associated with vascular calcification or vessel wall thickening has also been described in some adults with ENPP1 Deficiency (Ferreira, Hackbarth, et al., 2021; Kotwal et al., 2020; Lorenz‐Depiereux et al., 2010). Variants in the *ENPP1* gene may also lead to OPLL, as demonstrated in mouse model studies (Nakamura et al., 1999) and patient case reports (Ferreira, Ansh, et al., 2022; Saito et al., 2011). These patients may suffer from nerve root compression resulting in radiculopathy and myelopathy, that may severely limit mobility and negatively affect patients'quality of life. One patient with OPLL due to ENPP1 Deficiency was originally diagnosed with diffuse idiopathic skeletal hyperostosis (DISH) before genetic testing (H. Kato et al., 2022). Findings of low/normal serum phosphorous with elevated FGF‐23 in some of these patients suggest that cervical ligament ossifications may represent a complication of ARHR2 (H. Kato et al., 2022; Saito et al., 2011).

While ENPP1 Deficiency is considered an autosomal recessive disorder, there are case reports of adults with monoallelic *ENPP1* variants who presented with early‐onset osteoporosis and fractures (Oheim et al., 2020). These patients experienced clinical features of low bone mineral density with biomarkers similar to biallelic ENPP1‐deficient patients, including elevated FGF23, phosphate wasting, and low PPi levels‐ described as intermediate with respect to biallelic ENPP1 Deficiency. The later onset of low bone mass and osteoporosis juxtaposed to biallelic ENPP1 Deficiency with clinically‐apparent rickets during childhood provides an intriguing possibility of a gene dosage effect in the presentation of skeletal complications of ENPP1 Deficiency (Oheim et al., 2020). Another patient with spinal ligament ossification and presumptive diagnosis of DISH was later found to have a heterozygous *ENPP1* variant, extending the association of ENPP1 variants into patients presenting with spinal ligament ossification (H. Kato et al., 2022).

## 4 *ENPP1* GENOTYPES

Among the 108 unique genotypes, 77 were identified from the literature search and an additional 31 unique genotypes identified from the natural history data or NIH clinic. There were several specific genotypes that were found across multiple individual patients. The most prevalent genotype identified was p.Pro305Thr/p.Pro305Thr, found in 5.8% (9/154) of all patients. This genotype was found among 5 families. Other frequently encountered genotypes include p.Gly186Arg/p.Gly186Arg (2.6%; 4/154) and p.Pro250Leu/p.Pro250Leu (2.6%; 4/154). Genotypes p.Tyr901Ser/p.Tyr901Ser, p.Lys905Alafs*16/p.Lys905Alafs*16, and p.Gly266Val/p.Gly266Val were each encountered in 1.9% of patients (3/154). An additional 1.9% of patients were homozygous for a large partial gene deletion removing the 3' portion of the *ENPP1* gene beginning in intron 23 and truncating the C‐terminal portion of the nuclease domain (c.2444+702_*868del/c.2444+702_*868del) encoded in exons 24 and 25. A total of 24 of the 108 unique genotypes were found in 2 patients each (48/154) and 77 genotypes were found only in one individual. Among the 31 genotypes found in multiple patients, 13 unique genotypes were discovered in at least 2 unrelated patients.

When categorizing the genotypes of the GACI/ARHR2 patients by zygosity pattern, 40.3% (62/154) were homozygous for *ENPP1* variants and 52.6% (81/154) were compound heterozygous. Finally, 7.1% (11/154) of the GACI/ARHR2 patients were heterozygous for *ENPP1* variants, 9/11 patients with variants deemed to be pathogenic or likely pathogenic.

Interestingly, one heterozygous patient who was diagnosed with GACI was found to have the benign *ENPP1* p.Arg821His polymorphism (Patel et al., 2004; Stella et al., 2016) in addition to the *ABCC6* variant p.Arg1314Trp. This *ABCC6* variant is a well‐characterized pathogenic variant having been found in at least 2 families with demonstrated segregation (Ferreira, Hackbarth, et al., 2021; Li et al., 2014) or otherwise in multiple patients within a large cohort (Legrand et al., 2017; Nitschke et al., 2012; Pfendner et al., 2007; Ramsay et al., 2009) and confirmed to be functionally consequential in multiple empirical studies (Le Saux et al., 2011; Letavernier et al., 2018; Pomozi et al., 2014; Pomozi, Brampton, Szeri, et al., 2017; Pomozi, Brampton, van de Wetering, et al., 2017; Ran & Thibodeau, 2017). The significance of this finding for the interrelatedness of the *ENPP1* and *ABCC6* pathways in disease causation is unclear. It is possible that the absence of biallelic *ENPP1* variants in these cases is because of true haploinsufficiency leading to disease or otherwise reflects a false negative result due to limitations of the sequencing technique applied in these studies. Additionally, two homozygous GACI patients harbored one *ABCC6* variant each; p.Arg1114Cys and p.Val514Ile.

## 5 ENPP1 VARIANTS

**Figure 2:**
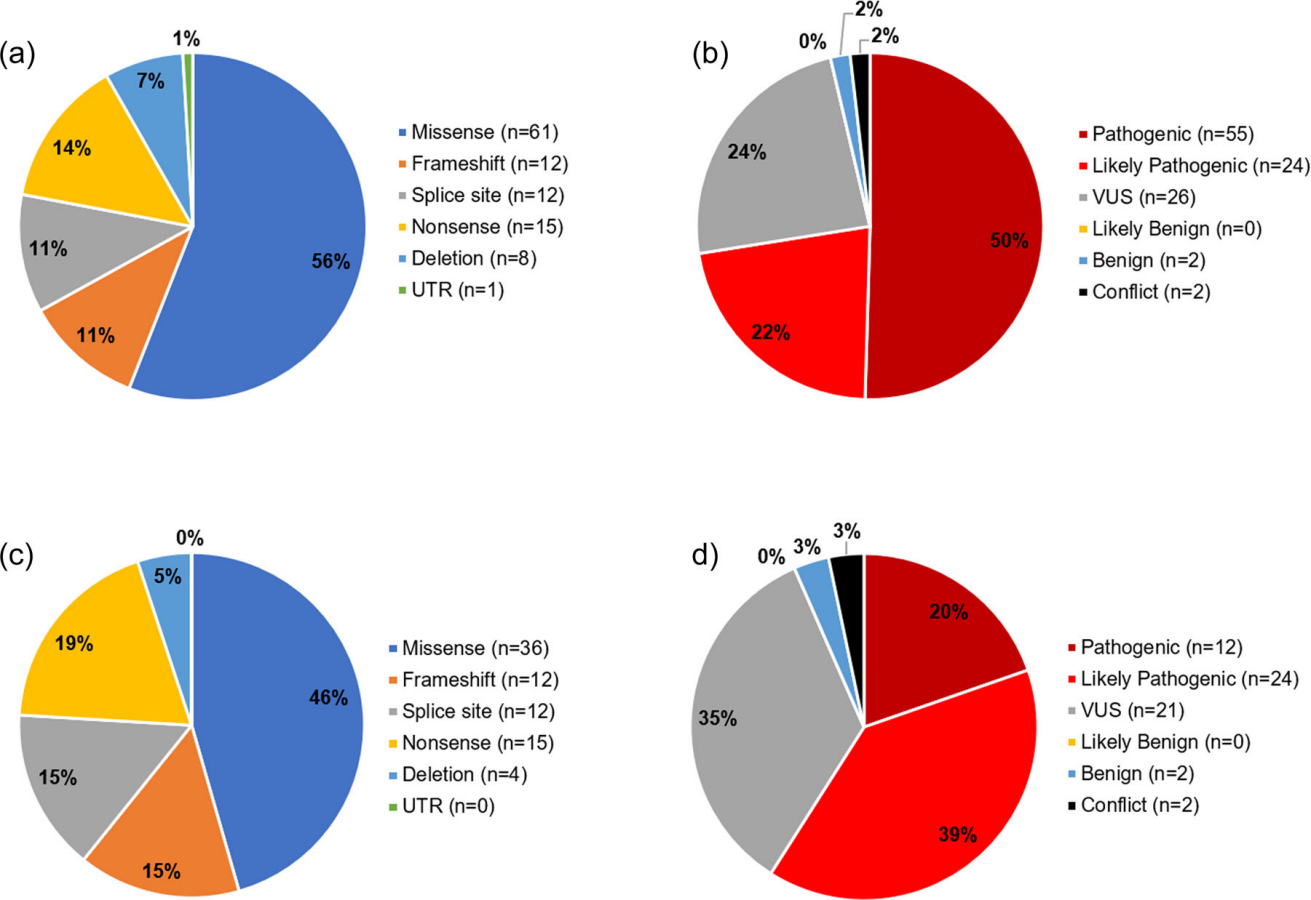
Characterization of *ENPP1* variants by type and pathogenicity call. (a) Relative prevalence of type of variant across all unique *ENPP1* variants. (b) Variant interpretation for all unique *ENPP1* variants. (c) Relative prevalence of type of variant for pathogenic or likely pathogenic *ENPP1* variants. (d) Variant interpretation for missense *ENPP1* variants. ENPP1, ectonucleotide pyrophosphatase/phosphodiesterase family member 1

Of the 109 unique *ENPP1* variants discovered in patients, 56.0% (61/109) were missense mutations, Figure 2a. Seperately, we found 72.5% were demonstrably disease‐causing based on the aggregated and interpreted evidence (55/109 pathogenic and 24/109 likely pathogenic). A smaller percentage (23.9%; 26/109) of these variants had insufficient evidence according to ACMG guidelines to draw definitive conclusions and were deemed VUS. Two variants were benign (1.8%; 2/109), and an additional two variants has conflicting evidence of pathogenicity (1.8%; 2/109). The number of variants across patients who had sufficient evidence to determine pathogenicity is depicted in Figure 2b. The presence of *ENPP1* VUS in patients with clinical evidence of GACI/ARHR2 suggests that these are in fact disease causing variants. Continual accumulation and characterization of such cases is critical to ensure appropriate diagnoses for all future GACI/ARHR2 patients considering the clinical presentation, laboratory values and an accurate assessment of the strength of a complete set of genetic evidence such as is typified in this study.

Among the 79 variants designated to be disease‐causing (pathogenic or likely pathogenic), 45.6% were missense (36/79) and 54.4% (43/79) were among the loss‐of‐function category including splice, nonsense, deletions, and frameshift. These results are depicted in Figure 2c. Among the missense variants, 59.0% (36/61) variants were likely pathogenic or pathogenic, and 34.4% were VUS (21/61). These results are depicted in Figure 2d.

When examining the distribution of these variants across the ENPP1 protein and the corresponding domains, 53.2% (58/109) were found within the phosphodiesterase domain and 30.3% (33/109) were found in the nuclease domain. These percentages did not significantly change when only the pathogenic and likely pathogenic variants were considered (51.9% and 32.9%, respective to the two previous percentages). It is important to consider whether this distribution is likely to have occurred simply by chance given the large size of these two domains which together comprise 43.4% (spanning amino acid 191–591 of the total 925 amino acids in ENPP1 as defined in UniProt (Bairoch & Apweiler, 1997), and 30.4% (spanning amino acids 654–925) by amino acid count. However, this calculation is focused on the unique variants alone and does not account for the differential prevalence of the individual variants across all alleles identified in this study.

**Figure 3:**
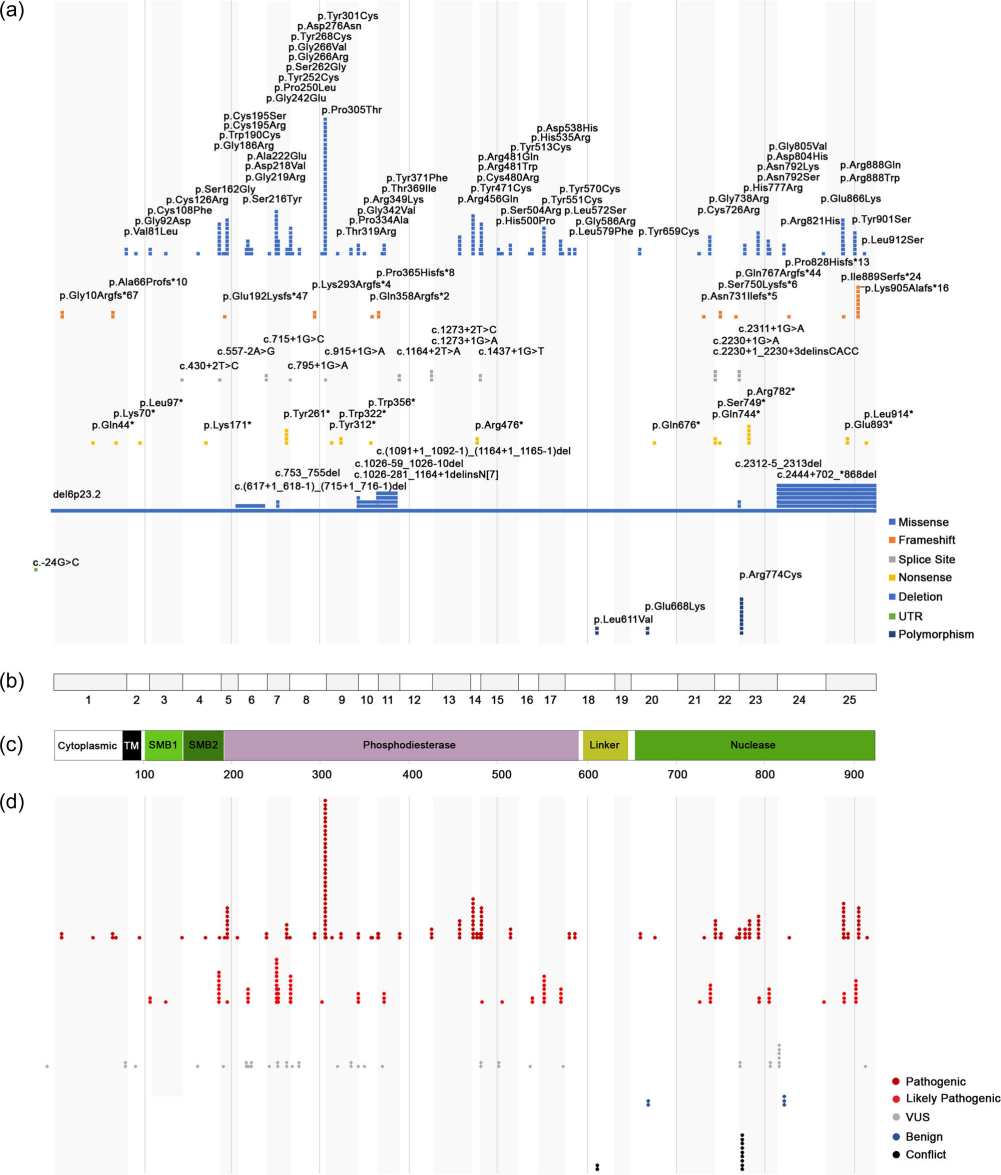
Distribution of unique variants across ENPP1 domains. (a) Depiction of total unique variants based on prevalence among patients in this study. (b) *ENPP1* exon structure. (c) ENPP1 structural domains. (d) Depiction of pathogenic, likely pathogenic and VUS variants as identified in sequencing results per patient. ENPP1, ectonucleotide pyrophosphatase/phosphodiesterase family member 1

We, therefore, next sought to better understand the distribution of these variants across the domain structure of the ENPP1 protein based not solely on the list of unique variants but rather based on their appearance across all alleles. Figure 3a depicts the distribution of all alleles across the ENPP1 linear protein structure based on the variant type relative to the exon structure of the fully spliced canonical ENPP1 mRNA transcript NM_006208.3 and the canonical ENPP1 protein sequence with its functional domains (NP_006619.2 isoform). These regions are depicted in Figure 3b,c, respectively. Based on this analysis of 308 patient alleles, we identified an incidence of 178 phosphodiesterase and 102 nuclease domain variants, suggesting a more significant selection bias for damaging effects to these domains leading to GACI and/or ARHR2 phenotypes but a small one. This result should underscore the conclusion that variants in other domains of ENPP1 should not be discounted. Indeed, 29 alleles containing *ENPP1* variants fell outside of the phosphodiesterase and nuclease domains including 14 pathogenic and likely pathogenic alleles in the two somatomedin B domains (SMB1 and SMB2). Seven alleles harboring nonsense or frameshift variants were found within the cytoplasmic and transmembrane regions. The variant pathogenicity designation for these and all other variants is depicted in Figure 3d based on the number of patients who were identified as having any specific variant. These results are displayed as pathogenic or likely pathogenic variants or otherwise with VUS, conflicting or benign.

Overall, there were 109 unique *ENPP1* variants found in patients. These variant data are shown in Table 2 as individual variants with the recurrence of each across all 154 patients indicated. The following variants each appeared in more than 5% of patients: p.Pro305Thr found in 15.6% of patients (24/154), p.Arg774Cys found in 6.5% (10/154), p.Tyr471Cys found in 6.5% (10/154), p.Arg888Trp found in 5.8% of patients (9/154), and p.Arg481Trp found in 5.2% of patients (8/154). Strikingly, 69.7% (76/109) of these unique variants were each recorded in a single patient or family. These 76 variants are, therefore, considered private, found in single families or a small number of individuals.

**Table 2:** *ENPP1* variants found in GACI and/or ARHR2 patients

Type	gDNA (NC_000006.12)	cDNA (NM_006208.3)	Protein (NP_006199.2)	Exon/Intron	ENPP1 Domains	ACMG Call	Recurrence of allele in this study
missense	g.131860504C>A	c.913C>A	p.Pro305Thr	8	Phosphodiesterase	Pathogenic	33
missense (polymorphism)	g.131884939C>T	c.2320C>T	p.Arg774Cys	23	Nuclease	Conflict	12
missense	g.131858701C>T	c.749C>T	p.Pro250Leu	7	Phosphodiesterase	Likely Pathogenic	11
missense	g.131872076A>G	c.1412A>G	p.Tyr471Cys	14	Phosphodiesterase	Pathogenic	10
missense	g.131890395C>T	c.2662C>T	p.Arg888Trp	25	Nuclease	Pathogenic	9
missense	g.131872926C>T	c.1441C>T	p.Arg481Trp	15	Phosphodiesterase	Pathogenic	8
frameshift	g.131890446_131890450del	c.2713_2717del	p.Lys905Alafs*16	25	Nuclease	Pathogenic	8
missense	g.131851267G>C	c.556G>C	p.Gly186Arg	4	SMB 2	Likely Pathogenic	8
missense	g.131852201T>C	c.583T>C	p.Cys195Arg	5	Phosphodiesterase	Pathogenic	8
missense	g.131875792A>G	c.1652A>G	p.Tyr551Cys	17	Phosphodiesterase	Likely Pathogenic	7
missense	g.131860388G>T	c.797G>T	p.Gly266Val	8	Phosphodiesterase	Likely Pathogenic	7
missense	g.131884994A>G	c.2375A>G	p.Asn792Ser	23	Nuclease	Pathogenic	6
deletion	g.131885765_131891379del	c.2444+702_*868del	p.?	Deletion of exons 23 and 25	Nuclease	VUS	6
missense	g.131890435A>C	c.2702A>C	p.Tyr901Ser	25	Nuclease	Likely Pathogenic	6
missense	g.131869451G>A	c.1367G>A	p.Arg456Gln	13	Phosphodiesterase	Pathogenic	5
missense	g.131882456G>A	c.2212G>A	p.Gly738Arg	21	Nuclease	Likely Pathogenic	5
nonsense	g.131884963C>T	c.2344C>T	p.Arg782*	23	Nuclease	Pathogenic	5
missense	g.131875849A>G	c.1709A>G	p.Tyr570Cys	17	Phosphodiesterase	Likely Pathogenic	4
missense	g.131885029G>C	c.2410G>C	p.Asp804His	23	Nuclease	Likely Pathogenic	4
missense	g.131854961A>T	c.653A>T	p.Asp218Val	6	Phosphodiesterase	Likely Pathogenic	4
missense	g.131858707A>G	c.755A>G	p.Tyr252Cys	7	Phosphodiesterase	Likely Pathogenic	4
nonsense	g.131858735C>G	c.783C>G	p.Tyr261*	7	Phosphodiesterase	Pathogenic	4
missense	g.131861704G>T	c.1025G>T	p.Gly342Val	9	Phosphodiesterase	Likely Pathogenic	3
missense	g.131864886A>T	c.1112A>T	p.Tyr371Phe	11	Phosphodiesterase	Likely Pathogenic	3
missense	g.131873023A>G	c.1538A>G	p.Tyr513Cys	15	Phosphodiesterase	Pathogenic	3
splice	g.131883775G>A	c.2311+1G>A	p.?	Splice donor between exons 22 and 23	Nuclease	Pathogenic	3
missense	g.131884949A>G	c.2330A>G	p.His777Arg	23	Nuclease	Pathogenic	3
missense	g.131886579G>A	c.2462G>A	p.Arg821His	24	Nuclease	Benign	3
missense	g.131890396G>A	c.2663G>A	p.Arg888Gln	25	Nuclease	Likely Pathogenic	3
deletion	g.(?−132186006)_(132186078‐?)del	c.(1091+1_1092‐1)_(1164+1_1165‐1)del	p.?	Deletion in between 10‐11 and 11‐12 removing exon 11	Phosphodiesterase	Pathogenic	2
missense	g.131861679C>G	c.1000C>G	p.Pro334Ala	9	Phosphodiesterase	VUS	2
deletion	g.132185365_132189157delinsN[7]	c.1026‐281_1164+1delinsN[7]	p.?	Deletion from intron 9 to 11 removing exons 10 and 11	Phosphodiesterase	Pathogenic	2
frameshift	g.131864868del	c.1094del	p.Pro365Hisfs*8	11	Phosphodiesterase	Pathogenic	2
splice	g.131864940T>A	c.1164+2T>A	p.?	Splice donor between exons 11 and 12	Phosphodiesterase	Pathogenic	2
splice	g.131868127G>A	c.1273+1G>A	p.?	Splice donor between exons 12 and 13	Phosphodiesterase	Pathogenic	2
nonsense	g.131872090C>T	c.1426C>T	p.Arg476*	14	Phosphodiesterase	Pathogenic	2
splice	g.131872102G>T	c.1437+1G>T	p.?	Splice donor between exons 14 and 15	Phosphodiesterase	Pathogenic	2
missense	g.131872923T>C	c.1438T>C	p.Cys480Arg	15	Phosphodiesterase	VUS	2
missense	g.131872984A>C	c.1499A>C	p.His500Pro	15	Phosphodiesterase	VUS	2
missense	g.131874314G>C	c.1612G>C	p.Asp538His	16	Phosphodiesterase	Likely Pathogenic	2
missense	g.131877005G>C	c.1737G>C	p.Leu579Phe	18	Phosphodiesterase	Pathogenic	2
missense	g.131877024G>A	c.1756G>A	p.Gly586Arg	18	Phosphodiesterase	Pathogenic	2
missense (polymorphism)	g.131877099C>G	c.1831C>G	p.Leu611Val	18	Linker 2	Conflict	2
frameshift	g.131808231_131808232del	c.196_197del	p.Ala66Profs*10	1	Cytoplasmic domain	Pathogenic	2
missense	g.131879910A>G	c.1976A>G	p.Tyr659Cys	20	Nuclease	Pathogenic	2
missense (polymorphism)	g.131879936G>A	c.2002G>A	p.Glu668Lys	20	Nuclease	Benign	2
splice	g.131882475_131882477delinsCACC	c.2230+1_2230+3delinsCACC	p.?	Deletion of splice donor site between exons 21 and 22	Nuclease	Pathogenic	2
nonsense	g.131882474C>T	c.2230C>T	p.Gln744*	21	Nuclease	Pathogenic	2
frameshift	g.131883711dup	c.2248dup	p.Ser750Lysfs*6	24	Nuclease	Pathogenic	2
deletion	g.131884926_131884932del	c.2312‐5_2313del	p.?	Deletion of splice site acceptor at exon 23	Nuclease	VUS	2
missense	g.131884995T>A	c.2376T>A	p.Asn792Lys	23	Nuclease	Likely Pathogenic	2
missense	g.131885033G>T	c.2414G>T	p.Gly805Val	23	Nuclease	VUS	2
missense	g.131847776G>T	c.241G>T	p.Val81Leu	2	Transmembrane region	VUS	2
nonsense	g.131890410G>T	c.2677G>T	p.Glu893*	25	Nuclease	Pathogenic	2
frameshift	g.131808061dup	c.26dup	p.Gly10Argfs*67	1	Cytoplasmic domain	Pathogenic	2
missense	g.131849999G>T	c.323G>T	p.Cys108Phe	3	SMB 1	Likely Pathogenic	2
missense	g.131854955C>A	c.647C>A	p.Ser216Tyr	6	Phosphodiesterase	VUS	2
missense	g.131854973C>A	c.665C>A	p.Ala222Glu	6	Phosphodiesterase	VUS	2
splice	g.131855024G>C	c.715+1G>C	p.?	splice acceptor between exons 6 and 7	Phosphodiesterase	Pathogenic	2
deletion	g.131858705_131858707del	c.753_755del	p.Tyr252del	7	Phosphodiesterase	VUS	2
missense	g.131858736A>G	c.784A>G	p.Ser262Gly	7	Phosphodiesterase	VUS	2
missense	g.131860417G>A	c.826G>A	p.Asp276Asn	8	Phosphodiesterase	VUS	2
frameshift	g.131860469_131860470del	c.878_879del	p.Lys293Argfs*4	8	Phosphodiesterase	Pathogenic	2
nonsense	g.131861645G>A	c.966G>A	p.Trp322*	9	Phosphodiesterase	Pathogenic	2
UTR	g.131808012G>C	c.−24G>C	p.?	1	Untranslated region	VUS	1
deletion	g.(?−132176066)_(132176163‐?)del	c.(617+1_618‐1)_(715+1_716‐1)del	p.?	Deletion in between exons 5‐6 and 6‐7 removing exon 6	Phosphodiesterase	Pathogenic	1
deletion	g.131864447_131864496del	c.1026‐59_1026‐10del	p.?	Intronic deletion between exons 9 and 10	Phosphodiesterase	VUS	1
missense	g.131864526G>A	c.1046G>A	p.Arg349Lys	9	Phosphodiesterase	VUS	1
nonsense	g.131864548G>A	c.1068G>A	p.Trp356*	10	Phosphodiesterase	Pathogenic	1
frameshift	g.131864552_131864562del	c.1072_1082del	p.Gln358Argfs*2	9	Phosphodiesterase	Pathogenic	1
missense	g.131864880C>T	c.1106C>T	p.Thr369Ile	11	Phosphodiesterase	VUS	1
deletion	g.131688637_132215008del	c.‐119399_*324497del	p.?	Entire gene	Whole gene	Pathogenic	1
splice	g.131868128T>C	c.1273+2T>C	p.?	Splice donor between exons 12 and 13	Phosphodiesterase	Pathogenic	1
nonsense	g.131808165C>T	c.130C>T	p.Gln44*	1	Cytoplasmic domain	Pathogenic	1
missense	g.131872927G>A	c.1442G>A	p.Arg481Gln	15	Phosphodiesterase	Likely Pathogenic	1
missense	g.131872995A>C	c.1510A>C	p.Ser504Arg	15	Phosphodiesterase	Likely Pathogenic	1
missense	g.131874306A>G	c.1604A>G	p.His535Arg	16	Phosphodiesterase	VUS	1
missense	g.131875855T>C	c.1715T>C	p.Leu572Ser	17	Phosphodiesterase	VUS	1
nonsense	g.131879960C>T	c.2026C>T	p.Gln676*	20	Nuclease	Pathogenic	1
nonsense	g.131808243A>T	c.208A>T	p.Lys70*	1	Cytoplasmic domain	Pathogenic	1
missense	g.131882420T>C	c.2176T>C	p.Cys726Arg	21	Nuclease	Likely Pathogenic	1
frameshift	g.131882436del	c.2192del	p.Asn731Ilefs*5	21	Nuclease	Pathogenic	1
splice	g.131882475G>A	c.2230+1G>A	p.?	Splice donor between exons 21 and 22	Nuclease	Pathogenic	1
nonsense	g.131883709C>G	c.2246C>G	p.Ser749*	22	Nuclease	Pathogenic	1
frameshift	g.131883763del	c.2300del	p.Gln767Argfs*44	22	Nuclease	Pathogenic	1
missense	g.131886713G>A	c.2596G>A	p.Glu866Lys	24	Nuclease	Likely Pathogenic	1
frameshift	g.131890397del	c.2664del	p.Ile889Serfs*24	25	Nuclease	Pathogenic	1
missense	g.131890468T>C	c.2735T>C	p.Leu912Ser	25	Nuclease	VUS	1
nonsense	g.131890474T>A	c.2741T>A	p.Leu914*	25	Nuclease	Pathogenic	1
missense	g.131847810G>A	c.275G>A	p.Gly92Asp	2	Transmembrane region	VUS	1
nonsense	g.131847823del	c.288del	p.Leu97*	2	Transmembrane region	Pathogenic	1
missense	g.131850052T>C	c.376T>C	p.Cys126Arg	3	SMB 1	Likely Pathogenic	1
splice	g.131850108T>C	c.430+2T>C	p.?	3	SMB 1	Pathogenic	1
missense	g.131851195A>G	c.484A>G	p.Ser162Gly	4	SMB 2	VUS	1
nonsense	g.131851222A>T	c.511A>T	p.Lys171*	4	SMB 2	Pathogenic	1
splice	g.131852173A>G	c.557‐2A>G	p.?	Splice acceptor between exons 4 and 5	SMB 2	Pathogenic	1
missense	g.131852188G>T	c.570G>T	p.Trp190Cys	5	Linker 1	VUS	1
frameshift	g.131852192del	c.574del	p.Glu192Lysfs*47	5	Phosphodiesterase	Pathogenic	1
missense	g.131852201T>A	c.583T>A	p.Cys195Ser	5	Phosphodiesterase	Likely Pathogenic	1
missense	g.131858677G>A	c.725G>A	p.Gly242Glu	7	Phosphodiesterase	VUS	1
splice	g.131858748G>A	c.795+1G>A	p.?	Splice acceptor between exons 7 and 8	Phosphodiesterase	Pathogenic	1
missense	g.131860387G>A	c.796G>A	p.Gly266Arg	8	Phosphodiesterase	Likely Pathogenic	1
missense	g.131860394A>G	c.803A>G	p.Tyr268Cys	8	Phosphodiesterase	VUS	1
missense	g.131860493A>G	c.902A>G	p.Tyr301Cys	8	Phosphodiesterase	Likely Pathogenic	1
splice	g.131860507G>A	c.915+1G>A	p.?	Splice donor between exons 8 and 9	Phosphodiesterase	Pathogenic	1
nonsense	g.131861615T>G	c.936T>G	p.Tyr312*	9	Phosphodiesterase	Pathogenic	1
missense	g.131861635C>G	c.956C>G	p.Thr319Arg	9	Phosphodiesterase	VUS	1
missense	g.131854963G>A	c.655G>A	p.Gly219Arg	6	Phosphodiesterase	VUS	1
frameshift	g.131886596_131886599dup	c.2479_2482dup	p.Pro828Hisfs*13	24	Nuclease	Pathogenic	1

Abbreviations: ARHR2, autosomal recessive hypophosphatemic rickets type 2; cDNA, complementary DNA; GACI, generalized arterial calcification of infancy; gDNA, genomic deoxyribonucleic acid; VUS, variants of uncertain significance.

### 5.1 Missense variants

Of the 36 pathogenic or likely pathogenic missense variants, 61.1% (22/36) fell within the phosphodiesterase region and 30.6% (11/36) fell within the nuclease domain (Figure 3a). These two domains are substantially larger than all other regions in this protein. The majority of the missense variants located in these two regions are pathogenic or likely pathogenic: 68.8% (11/16) of the nuclease‐containing and 59.5% (22/37) of the phosphodiesterase‐containing missense variants. The somatomedin B (SMB) 1 and 2 domains each harbored 2 missense variants. Both SMB1 missense variants are either pathogenic or likely pathogenic, and one of the SMB2 variants is likely pathogenic. The transmembrane domain contained 2 missense variants and the linker 1 region contained one. All three of these variants among these two smaller protein regions are classified as VUS.

To gain more insight into the relative prevalence of missense that are likely to be seen in diagnostic laboratories, we also considered the distribution of each variant type across the multiple different genotypes for the total number of patients identified in this work. This analysis revealed that across the 154 patients, 82.5% (127/154) had at least one missense variant and (54.5%) 84/154 had two missense variants. This high prevalence of missense variants in GACI/ARHR2 patients underscores the benefit of documenting and disseminating evidence about their pathogenicity. Additionally, there are several missense variants located in the distal portion of the nuclease region as well including p.Tyr901Ser (deemed likely pathogenic) and p.Leu912Ser (deemed VUS). Careful attention to these very distal 3' variants should be paid when interpreting novel variants.

### 5.2 Nonsense and frameshift variants

Forty‐five deletion, nonsense, frameshift, or splice variant are predicted loss of function (LoF) variants. All but five of the predicted LoF variants (including splice alterations, nonsense, frameshift, and deletion variants) were located in extracellular domains. These five include variants p.Gly10Argfs*67, p.Ala66Profs*10, and p.Lys70*, and p.Gln44* located in the cytoplasmic region and the p.Leu97* variant which locates in the C‐terminal end of the transmembrane region which spans residues 77‐97. All predicted LoF variants were deemed pathogenic, except for c.2444+702_*868del and c.2312‐5_2313del which are VUS. It is interesting to note the sizeable proportion of these LoF variants that appear at the distal C‐terminus of the ENPP1 protein removing sometimes only a fraction of the nuclease functional domain. The most extreme such examples include the frameshift variant p.Lys905Alafs*16 and nonsense variant p.Leu914*.

### 5.3 Splice variants

Twelve canonical splice site variants (11.0%, 12/109) were reported, all predicted to be pathogenic. As depicted in Figure 3a, seven of these are in the phosphodiesterase region, three in the nuclease‐like domain, and one in each of the SMB domains.

### 5.4 Indels and large deletions

Whole gene, exon or partial exon deletions or indels comprised 8.3% (9/109) of total variants. These included a single small intronic deletion within intron 9 near the acceptor site but not directly affecting the splice site itself (c.1026‐59_1026‐10del); two deletion variants affecting splice sites including a delins variant at the donor splice site of exon 21 within intron 21 (c.2230+1_2230+3delinsCACC) and a slightly larger deletion removing nucleotides at the acceptor side near exon 23 within intron 22 (c.2312‐5_2313del). Five larger deletions included one microdeletion affecting 6p23.2, and four deletions removing 1–2 exons each. Additionally, one in‐frame deletion was found; c.753_755del, resulting in p.Tyr252del. All of the deletions and indels lie within the phosphodiesterase or nuclease domain.

### 5.5 Polymorphisms

Two *ENPP1* variants were deemed benign polymorphisms (p.Glu668Lys and p.Arg821His) and two were found to have conflicting evidence of pathogenicity with insufficient evidence to make either a benign or a pathogenic designation. These include the p.Leu611Val variant and the polymorphism p.Arg774Cys. The latter p.Arg774Cys variant was found in 3.3% of alleles in gnomAD (as common as 9.7% in the Finnish population) and was, therefore, deemed too common to be disease‐causing according to clinical guidelines. However, at least one functional study examined the effect of p.Arg774Cys in SaOS2 osteosarcoma cell lines and showed 30‐40% of NPP activity indicating a possible damaging effect (Rutsch et al., 2003). A separate study in HEK293 cells showed no effect on PPi generation and localization resulting from this variant (Stella et al., 2016). The true nature of the effect of this variant on GACI or ARHR2 remains unclear. The less frequently encountered p.Leu611Val variant is present in gnomAD at 0.75% (and as commonly as 4.9% in African‐Americans) in an unselected population as well as being predicted to be functionally inert by SIFT, PolyPhen and MutationTaster. However, in a study of a family with the complex genotype p.Gln792Ser‐p.Glu668Lys‐p.Leu611Val, functional testing of NPP activity for SaSO2 osteosarcoma cell lines bearing the missense variant p.Glu668Lys were normal and the patient's phenotype was therefore attributed to the p.Leu611Val variant (Rutsch et al., 2003). Given that there were several affected patients with heterozygous *ENPP1* variants, it is unclear whether this interpretation is accurate. Based on these findings overall, the p.Arg774Cys and p.Leu611Val variants should, therefore, be interpreted cautiously within the context of the patient's overall clinical presentation and molecular results.

### 5.6 *ENPP1* variants in ClinVar

We next sought to determine whether any *ENPP1* variants not identified in our approach were catalogued in ClinVar. In total there were 341 *ENPP1* variants described in ClinVar at the time of publication. The majority of the ClinVar variants were classified as benign (37.2%; 127/341), likely benign (12.0%; 41/341) or otherwise VUS (34.0%; 116/341) with many of them being in the untranslated region (37.8%; 129/341), in deep intronic regions (32.6%; 111/341) or otherwise synonymous (6.5%; 22/341). The clinical importance of these variants is unclear as the majority lack corroborating evidence (93.3%; 318/341) with most having no reference citations (71.0%; 242/341), or otherwise only citing interpretation guidelines (20.2%; 69/341).

**Figure 4:**
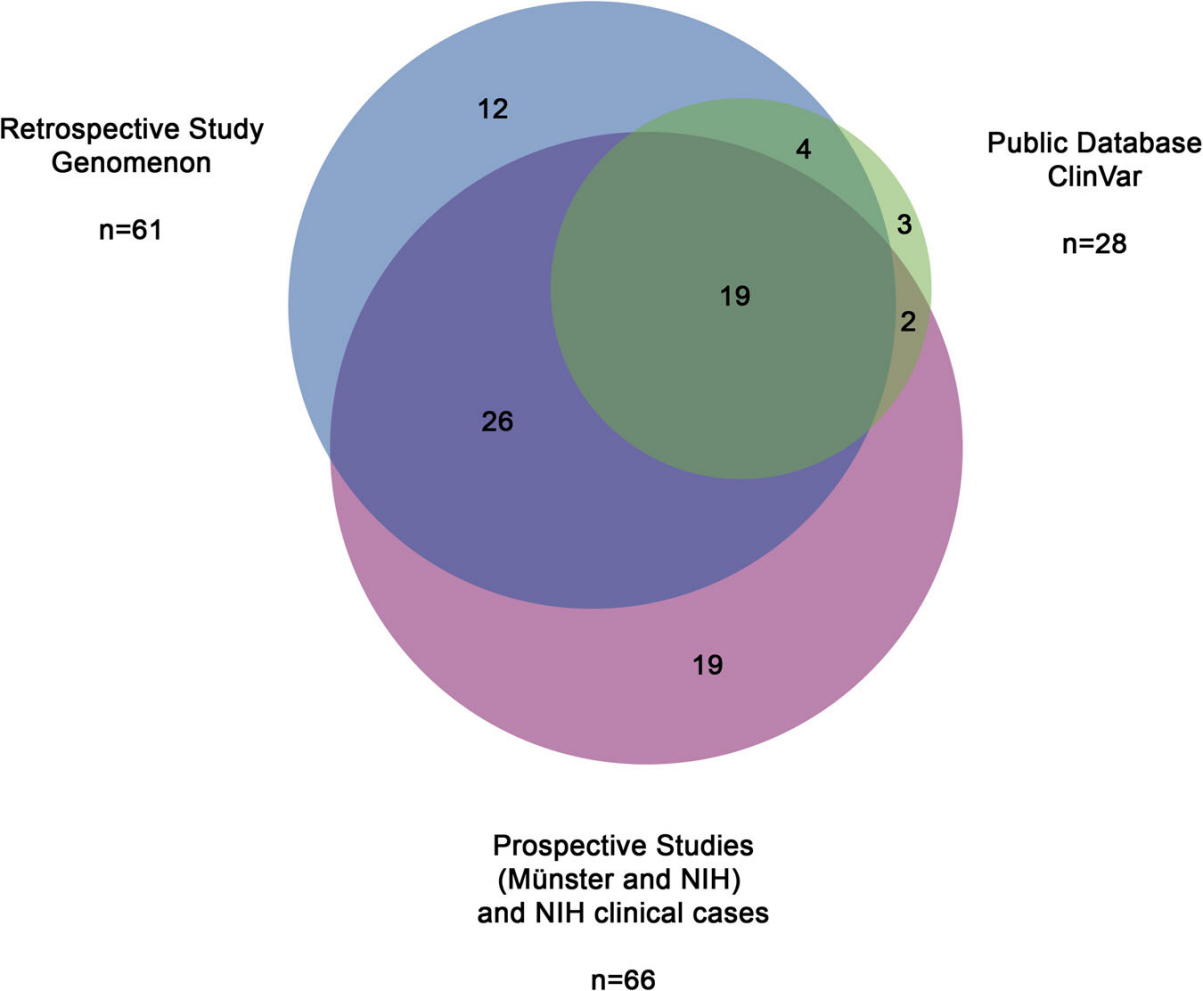
Source of pathogenic/likely pathogenic *ENPP1* variants including comprehensive retrospective analysis of published clinical and functional evidence performed by Genomenon, prospective information obtained in either of the two longitudinal natural history studies performed at the University Children's Hospital Münster or the NIH or otherwise identified in ClinVar. ENPP1, ectonucleotide pyrophosphatase/phosphodiesterase family member 1; NIH, National Institutes of Health

A summary of the source of *ENPP1* variants deemed to be pathogenic or likely pathogenic for GACI/ARHR2 is depicted in Figure 4. From ClinVar, 28 such variants were identified and from the Münster and NIH studies 61 and 66 variants were identified, respectively. The identification of 85 pathogenic/likely pathogenic variants including 56 variants not characterized in ClinVar represents a 3.0x increase in the number of variants in this study compared to ClinVar each with a thoroughly documented and evidence‐cited association with disease‐causation. There were three variants deemed pathogenic or likely pathogenic and associated with GACI and/or ARHR2 in ClinVar which were not characterized in this study.

## 6 IMPACT OF VARIANTS ON ENPP1 FUNCTION

The majority of variants in patients with ENPP1 Deficiency were located in the phosphodiesterase (PDE) or nuclease domains. The nuclease domain is tightly associated with the PDE domain and plays an important role in supporting it's catalytic activity (K. Kato et al., 2012). Truncating variant(s) in either of these domains, therefore, are expected to abolish ENPP1 enzymatic activity, resulting in decreased plasma PP_i_ and pathogenic calcification.

Over half of the individuals harbored two missense variants, which raises to question the mechanism of missense variant pathogenicity. Jansen et al. (2012) analyzed the location and stability of the ENPP1 protein product associated with disease‐associated *ENPP1* missense variants. Of the 26 missense variants located in the PDE or nuclease domain, the majority were predicted to cause protein destabilization (e.g., buried within cell membrane) or aggregation (Jansen et al., 2012). A study by Stella et al. further explored the impact of pathogenic missense variants on ENPP1 activity and PP_i_ generation (Stella et al., 2016). Of the 13 analyzed missense variants, eight showed complete loss of enzyme activity with no PP_i_ generation, four demonstrated reduced ENPP1 activity, and one variant had normal activity. Consistent with prior evidence, 5/8 of the missense variants with abolished ENPP1 activity were not localized in the plasma membrane. While the precise mechanism is still not fully elucidated, it has been hypothesized that loss of structural stability associated with missense variants may lead to protein misfolding, and reduced ENPP1 abundance (Jansen et al., 2012).

In addition to three LoF variants, four pathogenic or likely pathogenic missense variants in the SMB domains were identified in this review. Prior studies suggest that the SMB domains of ENPP1 are implicated in protein homodimerization (Bellacchio, 2012; Gijsbers et al., 2003), but do not interact with the catalytic domain (K. Kato et al., 2012). Of note, heterozygous variants in the SMB2 domain of ENPP1 underlie the autosomal‐dominant skin disorder, Cole disease, which is characterized by abnormal keratinization and hypopigmentation (Eytan et al., 2013).

Our study identified four variants located in the cytosolic domain, all of which were LoF variants deemed pathogenic. The cytosolic domain contains a di‐leucine motif (residues 49–50) that supports localization of ENPP1 to the basolateral surface, the region of the plasma membrane which buds off into matrix vesicles (Bello et al., 2001; Vaingankar et al., 2004). Mutations to one or both leucines were shown to direct ENPP1 expression to the apical surface. Functionally, these *ENPP1* mutants had approximately 50% lower matrix‐vesicle associated PP_i_ levels and increased calcification, as compared to wild‐type osteoblastic cells (SaOS‐2) (Vaingankar et al., 2004). Thus, while cytosolic *ENPP1* variants are uncommon, there is evidence supporting their potential for pathogenicity.

Other catalytically‐independent functions of ENPP1 which have been proposed through in‐vitro studies include prevention of osteoblast differentiation (Nam et al., 2011) and modulation of FGF‐23 expression (Mackenzie et al., 2012; Maulding et al., 2021).

## 7 ENPP1 DEFFICIENCY PHENOTYPES

Of the 154 patients identified, 99 (64.3%) were diagnosed with GACI, 29 were diagnosed with ARHR2 with no GACI (18.8%) and the remaining 26 (16.9%) were diagnosed with both phenotypes. These diagnoses were derived from the published literature or patients' medical charts, and thus it is difficult to ascertain if ARHR2‐only patients did not have early evidence of a GACI phenotype or when a thorough diagnostic workup was undertaken. Only a few of these patients reported undergoing imaging for the presence of vascular involvement, and arterial calcification or carotid artery stenosis was retrospectively identified in some of these patients.

**Figure 5:**
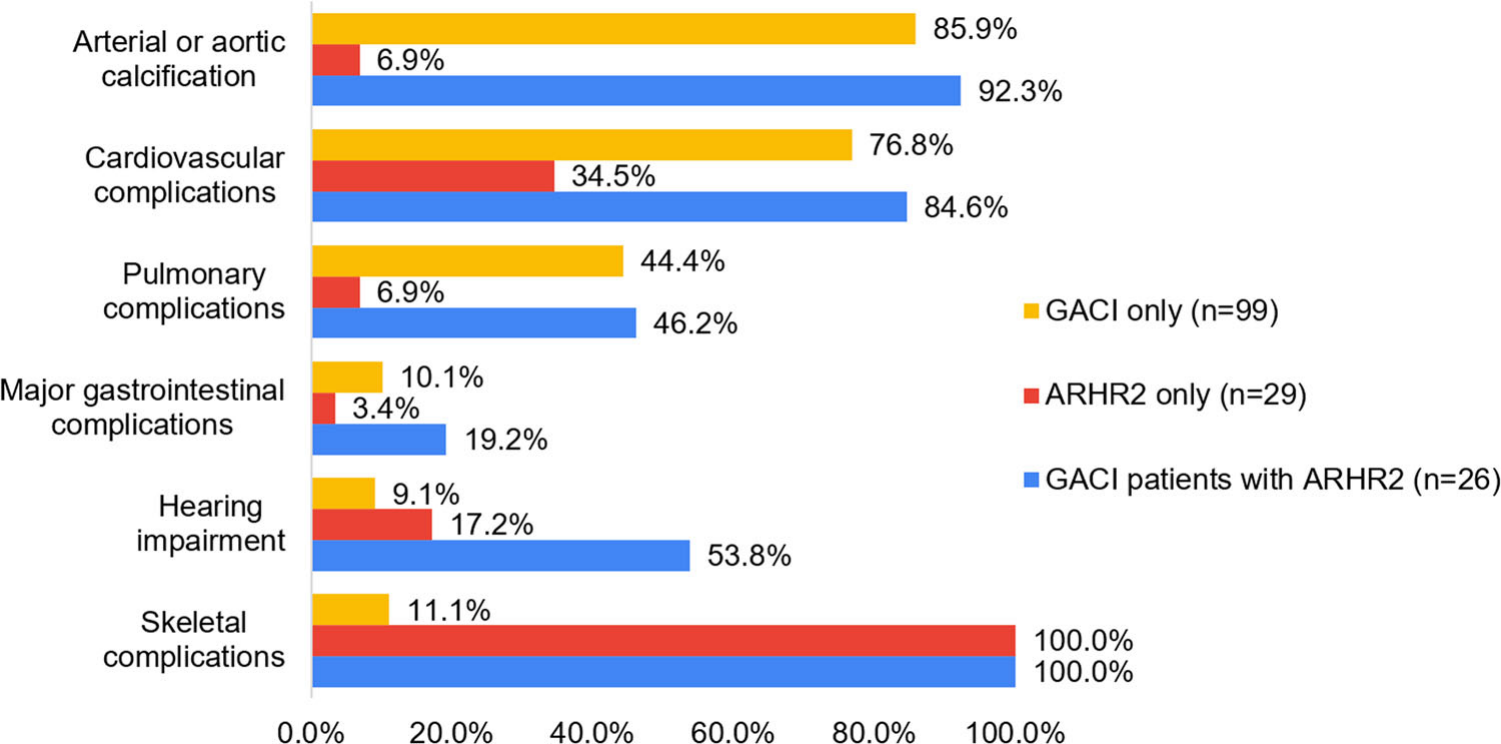
Prevalence of common phenotypes across patients with GACI only, ARHR2 only, and both GACI and ARHR2. ARHR2, autosomal recessive hypophosphatemic rickets type 2; GACI, generalized arterial calcification of infancy

A total of 125 patients were diagnosed with GACI (with or without ARHR2): 23 were diagnosed antenatally, 56 were diagnosed in the neonatal period, 7 were diagnosed in infancy, 2 were diagnosed in childhood, and 37 had an unknown age of diagnosis. Of the 120 GACI patients with a verifiable survival outcome, 65 (54.2%) died within the first 6 months of age. The most frequent phenotype reported in the 125 patients diagnosed with GACI was arterial or aortic calcification present in 87.2% of patients (85.9% of GACI patients without ARHR2 (85/99) and 92.3% of GACI patients with ARHR2 (24/26)) (Figure 5). Only two of the ARHR2‐only patients who did not have GACI showed arterial or aortic calcification (6.9%, 2/29). CV complications were reported in 76.8% (76/99) GACI‐only patients, 84.6% (22/26) in GACI and ARHR2 patients, and 34.5% (10/29) in ARHR2‐only patients. Pulmonary complications were also more common among GACI patients. Hearing impairment was most prevalent among patients diagnosed with both phenotypes (53.8%), followed by ARHR2‐only patients (17.2%) compared to GACI‐only (9.1%).

## 8 GENOTYPE‐PHENOTYPE CORRELATION

### 8.1 Phenotypes associated with the most common ENPP1 genotypes

Of the 9 patients with the genotype p.Pro305Thr/p.Pro305Thr, eight (89%) experienced early mortality (1 was stillborn, 5 died in the neonatal period, and 2 died in infancy). Arterial or aortic calcification was reported in all eight of these patients, and six had CV complications including hydrops fetalis (*n* = 4), heart failure (*n* = 4), and pericardial effusion (*n* = 3). However, this genotype may not lead to a universally fatal phenotype. One previously unpublished patient with p.Pro305Thr/p.Pro305Thr was diagnosed with ENPP1 deficiency in the neonatal period with major gastrointestinal complications but no CV complications, and survived the critical period of infancy. This patient later developed clinical manifestations of rickets, including bowed extremities, metaphyseal irregularities and cupping.

The second most common genotype, p.Gly186Arg/p.Gly186Arg, found in four patients, was associated with neonatal mortality in all cases. Polyhydramnios was detected in all four patients prenatally. These infants exhibited heterogeneous multiorgan complications including arterial calcification (*n* = 3), heart failure (*n* = 3), respiratory distress (*n* = 3), encephalopathy (*n* = 3), ischemic stroke (*n* = 2), abnormal brain morphology (*n* = 2) and liver dysfunction (*n* = 2).

The three patients who were homozygous for the large partial deletion (c.2444+702_*868del/c.2444+702_*868del) showed some phenotypic similarities. All three patients presented with skeletal symptoms of ARHR2 (2 during childhood, 1 with unknown time of onset) including bowed extremities (*n* = 3), hypophosphatemia (*n* = 2) and rickets (*n* = 3). Though none had a medical history of GACI, one of these patients was found to have thickening of aortic valves on echocardiography, and a second had a reported heart valve defect.

Taken together, individuals with the same genotype had heterogeneous disease manifestations. Ultimately the small number of patients with the same *ENPP1* genotype limits the conclusions that can be drawn regarding phenotype‐genotype correlations.

### 8.2 Phenotypes associated with reported monoallelic heterozygous *ENPP1* variants

Significant phenotypic variability was observed among the 10 patients with a single reported *ENPP1* variant. Eight patients (80%) were diagnosed with GACI and two were diagnosed with ARHR2 only. Of the eight patients diagnosed with GACI, seven had arterial or aortic calcification, four had CV complications, and three had pulmonary complications. Six of the eight patients died before the first 6 months of life, each with a heterozygous pathogenic variant. One patient had an unknown survival outcome, and the other was reported to be alive at age 11 with no record of skeletal manifestations. Of the patients diagnosed with ARHR2 only, both exhibited metaphyseal changes and joint pain or stiffness, and one had severe bowing of the legs. Concomitant CV complications were reported in one patient, and the other reported evidence of postnatal arterial calcification and hypertension, though did not have a recorded diagnosis of GACI.

Attribution of these severe GACI phenotypes to monoallelic ENPP1 Deficiency and a possible association of a GACI diagnosis with high rate of mortality in infants is noteworthy but it should be taken with caution. Several reports show intermediate levels of PPi, ENPP1 activity and analytes associated with bone mineralization in monoallelic ENPP1 patients, supporting the possibility of an ENPP1 gene dosage effect when regulating bone mass (H. Kato et al., 2022; Kotwal et al., 2020; Oheim et al., 2020). This is not in line with what has been reported in infants with ENPP1 deficiency where extensive calcification, cardiac complications, and a high mortality rate in the first year of age has been explained in part to nearly undetectable levels of PPi and low ENPP1‐derived adenosine induced stenosis (Nitschke et al., 2018). Additionally, one would expect to see this mortality phenomenon replicated in heterozygous mouse models that recapitulate the human disease very well. Based on how our data was collected (literature focused on patients diagnosed with GACI and ARHR2) a reasonable explanation of our finding is potentially explained by the inability of genetic analysis to detect a second *ENPP1* variant due to noncoding variants involving introns, promoter or enhancer regions. Indeed, authors of one of these published patient cases explicitly note that they could not exclude the potential existence of another variant in deep intronic regions (Ruf, 2005). Another possibility is that of monoallelic expression, a tissue‐specific mechanism whereby only one of the alleles is transcribed. The fact that single heterozygous variants can be found in patients with a severe phenotype warrants further genomic studies to discover the underlying mechanism.

### 8.3 Phenotypes associated with combined *ENPP1* and *ABCC6* variants

All three patients with identified *ENPP1* variant(s) and an *ABCC6* variant were diagnosed with GACI, and manifested with arterial calcifications (*n* = 3), cardiomegaly (*n* = 3) and/or left ventricular hypertrophy (*n* = 2). The two patients with homozygous, likely pathogenic *ENPP1* variants and an *ABCC6* variant both died—one in utero and the other at the age of 10. The latter also had significant skeletal and joint morbidities, and was wheelchair bound. The third patient had a single benign *ENPP1* variant in addition to an *ABCC6* variant, and was reported to be alive at 2.7 years of age.

### 8.4 Correlation between *ENPP1* genotype and survival outcome

An analysis of individual survival outcome by the number of truncating versus nontruncating *ENPP1* variants was performed and revealed no significant correlations (Table 3). Of the 147 patients with a published survival outcome, 48.3% (*n* = 71) died (9 stillborn or died in utero, 43 during the neonatal period, 19 during infancy). Compared to the 76 patients reported to be alive at last follow‐up (“survivors”), the distribution of patients by number of truncating variants was very similar: 50% of survivors and 59.2% of nonsurvivors harbored two nontruncating variants, followed by 23.7% of survivors and 21.1% of nonsurvivors with a mixed genotype. Interestingly, among patients harboring two truncating variants (*n* = 24) the majority (*n* = 16) were reported to be alive at last follow‐up. While this analysis is limited by the number of patients with no published survival outcome (*n* = 7), results suggest that the severity of ENPP1 phenotype is not alone predicted by the number of loss‐of‐function *ENPP1* variants.

**Table 3:** Individual survival outcome stratified by number of truncating versus nontruncating *ENPP1* variants

	Two truncating variants	Mixed genotype (1 truncating + 1 nontruncating)	Two nontruncating variants^a^	Heterozygous (truncating)	Heterozygous (nontruncating)^b^
Total population (*N* = 154)	24 (15.6%)	35 (22.7%)	84 (54.5%)	3 (1.9%)	8 (5.2%)
Died (*n* = 71)	8 (11.3%)	15 (21.1%)	42 (59.2%)	2 (2.8%)	4 (5.6%)
Survived (*n* = 76)	16 (21.1%)	18 (23.7%)	38 (50.0%)	1 (1.3%)	3 (3.9%)
Unknown (*n* = 7)	0 (0%)	2 (28.6%)	4 (57.1%)	0 (0%)	1 (14.3%)

Abbreviation: ENPP1, ectonucleotide pyrophosphatase/phosphodiesterase family member 1.

a Includes two patients with homozygous nontruncating *ENPP1* variants, in addition to an *ABCC6* variant.

b Includes one patient with a single nontruncating *ENPP1* variant, in addition to an *ABCC6* variant.

## 9 DIAGNOSTIC STRATEGIES

Identifying high risk groups for whom genetic testing may help ensure an accurate diagnosis is crucial. The following patient types could benefit from gene sequencing with a panel including *ENPP1*. Brightness of great vessels on prenatal or neonatal ultrasound or echocardiogram warrant ENPP1 Deficiency evaluation (Ziegler et al., 1993). In utero, findings such as pericardial effusion, polyhydramnios, cardiomyopathy and nonimmune hydrops fetalis may be indicative of ENPP1 Deficiency, especially in combination with family history of early fetal death or rickets. An ENPP1 Deficiency diagnosis should also be considered for children with evidence of hypophosphatemic rickets, joint, ligamentous, or arterial calcification/stenosis, or idiopathic hearing loss. Adult patients have experienced significant delays in diagnosis, extending into the third, fourth and fifth decade of life in some instances. *ENPP1* genetic testing should be considered in adults with any of the following: osteomalacia or early‐onset osteoporosis with low fasting serum phosphorus and low ratio of the tubular maximum reabsorption of phosphate to glomerular filtration rate, tumor‐induced osteomalacia without evidence of tumor, *PHEX*‐negative XLH, joint calcification, joint stiffness secondary to enthesopathy, OPLL, diffuse DISH, or fusion of cervical vertebrae. Where testing is available, low plasma PPi may facilitate a diagnosis of ENPP1 Deficiency. Including the *ENPP1* gene in appropriate gene panels such as skeletal, pediatric stroke and congenital cardiac disorder panels will increase the diagnostic yield for the disease. Reports also suggest adults with monoallelic *ENPP1* variants exhibit low ENPP1 activity, low PPi levels and FGF23‐mediated hypophosphatemia, and for these patients presenting with low bone mineral density, early‐onset osteoprorosis, or presentation of OPLL and DISH, a diagnosis of ENPP1 Deficiency should be entertained.

Integration of the locus‐specific database (LSDB) for ENPP1 Deficiency into the Mastermind Genomic Search Engine used by clinical labs worldwide could increase diagnostic yield by providing clinical laboratories with direct access to reference‐cited pathogenicity classifications for every *ENPP1* variant. These classifications and their corresponding evidence, viewed through Mastermind, can be easily reviewed, validated, and integrated into these laboratories' existing workflows to enable more rapid and consistent interpretation of *ENPP1* variants, both within and across individual laboratories. This is especially true for novel variants, where evidence recorded for similar variants, including those on or around the same residue, can be utilized to produce a classification. Additionally, as this LSDB will be continually updated with newly published evidence, it can also be utilized for rapid reanalysis of previously identified variants, which has traditionally been a major challenge for clinical laboratories and can lead to missed diagnoses.

## 10 DISCUSSION AND FUTURE PROSPECTS

This review highlights the fact that among patients with a GACI or ARHR2 phenotype, approximately half of all *ENPP1* variants are located in the PDE domain, while about one‐third of variants are located in the nuclease domain; both of these domains are important for catalytic activity. Additionally, about half of all *ENPP1* disease‐causing variants are truncating, reinforcing the fact that the disease is a consequence of loss of ENPP1 function and enzyme activity, thus making it amenable to enzyme replacement therapy. Despite the high precent of mutations found in the domains in ENPP1 important for catalytic activity in patients diagnosed with GACI and ARHR2, characterized by progressive arterial and tissue calcifications, recent advances suggest the possibility of a noncatalytic role for ENPP1 in skeletal phenotype perhaps through the WNT signaling pathway (Maulding et al., 2021; Zimmerman et al., 2022). We note that our data focused on reported diagnosis of GACI or ARHR2 and it is possible that we have under‐reported the prevalence of pathogenic variants in noncatalytic domains of ENPP1, possibly specific to patients diagnosed with OPLL or early‐onset osteoporosis. More work is needed to elucidate the potential noncatalytic role of ENPP1 in GACI and ARHR2 patients.

We also found that some variants previously described as disease‐causing are too frequent to be considered pathogenic. As an example, the recurrent variant p.Arg774Cys (found in 12 total alleles, with 6.5% or 10/154 patients harboring one or two of this variant) has an allele frequency almost as high as 10% in certain populations. Similarly, the allele frequency of the p.Leu611Val variant is almost as high as 5% in certain populations. Although these two variants are unlikely to be pathogenic, it is still possible that they may lead to disease when in cis between each other or another common variant in combination with a rare pathogenic variant in trans in the other allele (monogenic triallelic inheritance).

Monoallelic inheritance has been associated with skeletal disease in the form of low bone mineral density, originally described as early‐onset osteoporosis as well as OPLL and DISH (H. Kato et al., 2022; Oheim et al., 2020; Saito et al., 2011). This phenotype has been ascribed to intermediate levels of PPi, and ENPP1 activity, supporting the possibility of an ENPP1 gene dosage effect when regulating bone mass, suggesting ENPP1 Deficiency may be autosomal dominant in specific states (H. Kato et al., 2022; Kotwal et al., 2020; Oheim et al., 2020). The mechanism by which monoallelic *ENPP1* variants leads to skeletal disease is still being elucidated. Anecdotally, parents of most patients with GACI or ARHR2 do not have clinically significant osteomalacia, although it is possible that this complication of monoallelic ENPP1 deficiency depends on the severity of enzyme deficiency associated with each variant, and that thus only single variants associated with severe enzyme deficiency will lead to skeletal disease. Therefore, one possibility is that haploinsufficiency (leading to approximately 50% residual enzyme activity) versus a dominant negative effect (leading to <50% enzyme activity) would be a possible mechanism for monoallelic ENPP1 deficiency; further work is needed to assess whether this is a valid hypothesis. Another possibility is that a second variant was missed in these adults, such as a noncoding variant in the promoter or deep‐intronic regions. Yet a third possibility is that common variants in trans with rare severe variants might lead to disease, but are not reported by clinical laboratories as they are assumed to be benign. As mentioned earlier, some common variants (p.Arg774Cys and p.Leu611Val) have been shown to lead to decreased PDE activity in SaOS2 osteosarcoma cell lines. It is thus possible that although these variants will not lead to disease in homozygosity, they might when associated with other variants in the other allele. There is precedent for such mechanism, as in partial biotinidase deficiency, where a common variant (with an allele frequency of 3.9% in a European non‐Finnish population, and up to 5.6% in a Finnish population) is not associated with disease in homozygosity, but leads to clinical manifestations in combination with a pathogenic variant in trans (Swango et al., 1998). Interestingly, we identified in the literature eight infants with a severe GACI phenotype and early mortality harboring with only a single heterozygous variant in *ENPP1*. This puts the gene dose effect at odds with severe vascular calcifications and high mortality observed in infants is largely ascribed to nearly undetectable PPi levels (Nitschke et al., 2018). How monoallelic ENPP1 Deficiency could result in a severe, highly fatal GACI phenotype is unclear, and merits further study as more GACI patients with a single *ENPP1* variant are identified. Evaluation of PPi levels in infants may help discern whether this is true ENPP1 haploinsufficiency, or if a second undetectable variant was likely present.

It is not known, however, whether the severity of enzyme deficiency is associated with a more severe phenotype. We found that 8/24 (33.3%) of patients with two truncating variants and known survival outcome succumbed to their disease, while mortality was somewhat higher among patients with known survival outcomes who harbored two nontruncating variants (42/71, or 59.2%). This appears to confirm the fact described in prior smaller cohorts asserting the lack of genotype‐phenotype correlation in ENPP1 Deficiency (Ferreira, Hackbarth, et al., 2021). It should also be noted that the extent to which phenotypes are described in the literature vary between authors. The relatively high prevalence of cardiovascular complications reported in patients with an ARHR2 only diagnosis (34.5%) affirms the understanding of ENPP1 Deficiency as a single spectrum of disease, and points to the importance of thorough cardiovascular workup and continued monitoring in children and adults. Therefore, the absence of a phenotype in literature‐sourced patient information may be limited to individual authors' descriptions or assessments, rather than a confirmed lack of the trait.

Though this is the largest study of GACI and ARHR2 patients ever gathered, the statistical significance of these findings is uncertain. We hope that this work will serve as a starting point for continued aggregation of genetic and phenotypic data as new GACI and ARHR2 patients are identified and characterized.

## CONFLICTS OF INTEREST

Stephanie A. Mercurio, Lauren Chunn, and Mark J. Kiel are full‐time employees of Genomenon, Inc. Gus Khursigara and Kathleen Wray are full‐time employees of Inozyme Pharma, Inc. and own stock in Inozyme Pharma. Ulrike Botschen and Frank Rutsch received funding from Inozyme Pharma. Carlos R. Ferreira reports a collaboration with Inozyme Pharma, Inc. as part of a Cooperative Research and Development Agreement.

## Supporting information

Supplementary information.

## References

[humu24477-bib-0001] Adzhubei, I. A. , Schmidt, S. , Peshkin, L. , Ramensky, V. E. , Gerasimova, A. , Bork, P. , Kondrashov, A. S. , & Sunyaev, S. R. (2010). A method and server for predicting damaging missense mutations. Nature Methods, 7(4), 248–249. 10.1038/nmeth0410-248 20354512 PMC2855889

[humu24477-bib-0002] Albayrak, G. , Silistreli, E. , Ergur, B. , Kalkan, S. , Karabay, O. , Erdal, A. C. , & Acikel, U. (2015). Inhibitory effect of adenosine on intimal hyperplasia and proliferation of smooth muscle cells in a carotid arterial anastomosis animal model. Vascular, 23(2), 124–131. 10.1177/1708538114533962 24803551

[humu24477-bib-0003] Albright, R. A. , Stabach, P. , Cao, W. , Kavanagh, D. , Mullen, I. , Braddock, A. A. , Covo, M. S. , Tehan, M. , Yang, G. , Cheng, Z. , Bouchard, K. , Yu, Z.‐X. , Thorn, S. , Wang, X. , Folta‐Stogniew, E. J. , Negrete, A. , Sinusas, A. J. , Shiloach, J. , Zubal, G. , … Braddock, D. T. (2015). ENPP1‐Fc prevents mortality and vascular calcifications in rodent model of generalized arterial calcification of infancy. Nature Communications, 6, 10006. 10.1038/ncomms10006 PMC4686714 26624227

[humu24477-bib-0004] Apschner, A. , Huitema, L. F. , Ponsioen, B. , Peterson‐Maduro, J. , & Schulte‐Merker, S. (2014). Zebrafish enpp1 mutants exhibit pathological mineralization, mimicking features of generalized arterial calcification of infancy (GACI) and pseudoxanthoma elasticum (PXE). Disease Models & Mechanisms, 7(7), 811–822. 10.1242/dmm.015693 24906371 PMC4073271

[humu24477-bib-0005] Bairoch, A. , & Apweiler, R. (1997). The SWISS‐PROT protein sequence database: Its relevance to human molecular medical research. Journal of Molecular Medicine, 75(5), 312–316. 9181472

[humu24477-bib-0006] Bellacchio, E. (2012). In silico analysis of the two tandem somatomedin B domains of ENPP1 reveals hints on the homodimerization of the protein. Journal of Cellular Physiology, 227(11), 3566–3574. 10.1002/jcp.24058 22262087

[humu24477-bib-0007] Bello, V. , Goding, J. W. , Greengrass, V. , Sali, A. , Dubljevic, V. , Lenoir, C. , Trugnan, G. , & Maurice, M. (2001). Characterization of a di‐leucine‐based signal in the cytoplasmic tail of the nucleotide‐pyrophosphatase NPP1 that mediates basolateral targeting but not endocytosis. Molecular Biology of the Cell, 12(10), 3004–3015. 10.1091/mbc.12.10.3004 11598187 PMC60151

[humu24477-bib-0008] Brachet, C. , Mansbach, A. L. , Clerckx, A. , Deltenre, P. , & Heinrichs, C. (2014). Hearing loss is part of the clinical picture of ENPP1 loss of function mutation. Hormone Research in Paediatrics, 81(1), 63–66. 10.1159/000354661 24216977

[humu24477-bib-0009] Cheng, Z. , O'Brien, K. , Howe, J. , Sullivan, C. , Schrier, D. , Lynch, A. , Jungles, S. , Sabbagh, Y. , & Thompson, D. (2021). INZ‐701 prevents ectopic tissue calcification and restores bone architecture and growth in ENPP1‐deficient mice. Journal of Bone and Mineral Research, 36(8), 1594–1604. 10.1002/jbmr.4315 33900645

[humu24477-bib-0010] Chunn, L. M. , Nefcy, D. C. , Scouten, R. W. , Tarpey, R. P. , Chauhan, G. , Lim, M. S. , Elenitoba‐Johnson, K. S. J. , Schwartz, S. A. , & Kiel, M. J. (2020). Mastermind: A comprehensive genomic association search engine for empirical evidence curation and genetic variant interpretation. Frontiers in Genetics, 11, 577152. 10.3389/fgene.2020.577152 33281875 PMC7691534

[humu24477-bib-0011] Dubey, R. K. , Gillespie, D. G. , Mi, Z. , & Jackson, E. K. (1998). Adenosine inhibits growth of human aortic smooth muscle cells via A2B receptors. Hypertension, 31(1 Pt 2), 516–521. 10.1161/01.hyp.31.1.516. 9453355

[humu24477-bib-0012] den Dunnen, J. T. , Dalgleish, R. , Maglott, D. R. , Hart, R. K. , Greenblatt, M. S. , McGowan‐Jordan, J. , Roux, A.‐F. , Smith, T. , Antonarakis, S. E. , & Taschner, P. E. M. (2016). HGVS recommendations for the description of sequence variants: 2016 update. Human Mutation, 37(6), 564–569. 10.1002/humu.22981 26931183

[humu24477-bib-0013] Eytan, O. , Morice‐Picard, F. , Sarig, O. , Ezzedine, K. , Isakov, O. , Li, Q. , Ishida‐Yamamoto, A. , Shomron, N. , Goldsmith, T. , Fuchs‐Telem, D. , Adir, N. , Uitto, J. , Orlow, S. J. , Taieb, A. , & Sprecher, E. (2013). Cole disease results from mutations in ENPP1. The American Journal of Human Genetics, 93(4), 752–757. 10.1016/j.ajhg.2013.08.007 24075184 PMC3791268

[humu24477-bib-0014] Ferreira, C. R. , Ansh, A. J. , Nester, C. , O'Brien, C. , Stabach, P. R. , Murtada, S.‐I. , Lester, E. R. , Khursigara, G. , Molloy, L. , Carpenter, T. O. , & Braddock, D. T. (2022). Musculoskeletal comorbidities and quality of life in ENPP1‐Deficient adults and the response of enthesopathy to enzyme replacement therapy in murine models. Journal of Bone and Mineral Research: The Official Journal of the American Society for Bone and Mineral Research, 37(3), 494–504. 10.1002/jbmr.4487 34882836 PMC9667476

[humu24477-bib-0015] Ferreira, C. R. , Hackbarth, M. E. , Ziegler, S. G. , Pan, K. S. , Roberts, M. S. , Rosing, D. R. , Whelpley, M. S. , Bryant, J. C. , Macnamara, E. F. , Wang, S. , Müller, K. , Hartley, I. R. , Chew, E. Y. , Corden, T. E. , Jacobsen, C. M. , Holm, I. A. , Rutsch, F. , Dikoglu, E. , Chen, M. Y. , … Gahl, W. A. (2021). Prospective phenotyping of long‐term survivors of generalized arterial calcification of infancy (GACI. Genetics in Medicine, 23(2), 396–407. 10.1038/s41436-020-00983-0 33005041 PMC7867608

[humu24477-bib-0016] Ferreira, C. R. , Kavanagh, D. , Oheim, R. , Zimmerman, K. , Stürznickel, J. , Li, X. , Stabach, P. , Rettig, R. L. , Calderone, L. , MacKichan, C. , Wang, A. , Hutchinson, H. A. , Nelson, T. , Tommasini, S. M. , Kroge, S. , Fiedler, I. A. , Lester, E. R. , Moeckel, G. W. , Busse, B. , … Braddock, D. T. (2021). Response of the enpp1‐deficient skeletal phenotype to oral phosphate supplementation and/or enzyme replacement therapy: Comparative studies in humans and mice. Journal of Bone and Mineral Research, 36(5), 942–955. 10.1002/jbmr.4254 33465815 PMC8739051

[humu24477-bib-0017] Ferreira, C. R. , Kintzinger, K. , Hackbarth, M. E. , Botschen, U. , Nitschke, Y. , Mughal, M. Z. , Baujat, G. , Schnabel, D. , Yuen, E. , Gahl, W. A. , Gafni, R. I. , Liu, Q. , Huertas, P. , Khursigara, G. , & Rutsch, F. (2021). Ectopic calcification and hypophosphatemic rickets: natural history of ENPP1 and ABCC6 deficiencies. Journal of Bone and Mineral Research, 36(11), 2193–2202. 10.1002/jbmr.4418 34355424 PMC8595532

[humu24477-bib-0018] Fleisch, H. , & Bisaz, S. (1962). Mechanism of calcification: Inhibitory role of pyrophosphate. Nature, 195, 911. 10.1038/195911a0 13893487

[humu24477-bib-0019] Gijsbers, R. , Ceulemans, H. , & Bollen, M. (2003). Functional characterization of the non‐catalytic ectodomains of the nucleotide pyrophosphatase/phosphodiesterase NPP1. Biochemical Journal, 371(Pt 2), 321–330. 10.1042/BJ20021943 12533192 PMC1223305

[humu24477-bib-0020] Goding, J. W. , Grobben, B. , & Slegers, H. (2003). Physiological and pathophysiological functions of the ecto‐nucleotide pyrophosphatase/phosphodiesterase family. Biochimica et Biophysica Acta (BBA) ‐ Molecular Basis of Disease, 1638(1), 1–19. 10.1016/s0925-4439(03)00058-9 12757929

[humu24477-bib-0021] Haffner, D. , Emma, F. , Eastwood, D. M. , Duplan, M. B. , Bacchetta, J. , Schnabel, D. , Wicart, P. , Bockenhauer, D. , Santos, F. , Levtchenko, E. , Harvengt, P. , Kirchhoff, M. , Di Rocco, F. , Chaussain, C. , Brandi, M. L. , Savendahl, L. , Briot, K. , Kamenicky, P. , Rejnmark, L. , & Linglart, A. (2019). Clinical practice recommendations for the diagnosis and management of X‐linked hypophosphataemia. Nature Reviews. Nephrology, 15(7), 435–455. 10.1038/s41581-019-0152-5 31068690 PMC7136170

[humu24477-bib-0022] Jansen, S. , Perrakis, A. , Ulens, C. , Winkler, C. , Andries, M. , Joosten, R. P. , Van Acker, M. , Luyten, F. P. , Moolenaar, W. H. , & Bollen, M. (2012). Structure of NPP1, an ectonucleotide pyrophosphatase/phosphodiesterase involved in tissue calcification, Structure (London, England: 1993). 20(11). 1948–1959. 10.1016/j.str.2012.09.001 23041369

[humu24477-bib-0023] Johnson, K. , Goding, J. , Van Etten, D. , Sali, A. , Hu, S.‐I. , Farley, D. , Krug, H. , Hessle, L. , Millán, J. L. , & Terkeltaub, R. (2003). Linked deficiencies in extracellular PP(i) and osteopontin mediate pathologic calcification associated with defective PC‐1 and ANK expression. Journal of Bone and Mineral Research, 18(6), 994–1004. 10.1359/jbmr.2003.18.6.994 12817751

[humu24477-bib-0024] Karczewski, K. J. , Francioli, L. C. , Tiao, G. , Cummings, B. B. , Alföldi, J. , Wang, Q. , Collins, R. L. , Laricchia, K. M. , Ganna, A. , Birnbaum, D. P. , Gauthier, L. D. , Brand, H. , Solomonson, M. , Watts, N. A. , Rhodes, D. , Singer‐Berk, M. , England, E. M. , Seaby, E. G. , Kosmicki, J. A. , … Salomaa, V. (2020). The mutational constraint spectrum quantified from variation in 141,456 humans. Nature, 581(7809), 434–443. 10.1038/s41586-020-2308-7 32461654 PMC7334197

[humu24477-bib-0025] Kato, H. , Ansh, A. J. , Lester, E. R. , Kinoshita, Y. , Hidaka, N. , Hoshino, Y. , Koga, M. , Taniguchi, Y. , Uchida, T. , Yamaguchi, H. , Niida, Y. , Nakazato, M. , Nangaku, M. , Makita, N. , Takamura, T. , Saito, T. , Braddock, D. T. , & Ito, N. (2022). Identification of ENPP1 haploinsufficiency in patients with diffuse idiopathic skeletal hyperostosis and early‐onset osteoporosis. Journal of Bone and Mineral Research, 37(6), 1125–1135. 10.1002/jbmr.4550 35340077 PMC9177665

[humu24477-bib-0026] Kato, K. , Nishimasu, H. , Okudaira, S. , Mihara, E. , Ishitani, R. , Takagi, J. , Aoki, J. , Nureki, O. 2012. Crystal structure of Enpp1, an extracellular glycoprotein involved in bone mineralization and insulin signaling. Proceedings of the National Academy of Sciences of the United States of America, 109(42), 16876–16881. 10.1073/pnas.1208017109 23027977 PMC3479499

[humu24477-bib-0027] Kotwal, A. , Ferrer, A. , Kumar, R. , Singh, R. J. , Murthy, V. , Schultz‐Rogers, L. , Zimmermann, M. , Lanpher, B. , Zimmerman, K. , Stabach, P. R. , Klee, E. , Braddock, D. T. , & Wermers, R. A. (2020). Clinical and biochemical phenotypes in a family with ENPP1 mutations. Journal of Bone and Mineral Research, 35(4), 662–670. 10.1002/jbmr.3938 31826312 PMC7771569

[humu24477-bib-0028] Legrand, A. , Cornez, L. , Samkari, W. , Mazzella, J.‐M. , Venisse, A. , Boccio, V. , Auribault, K. , Keren, B. , Benistan, K. , Germain, D. P. , Frank, M. , Jeunemaitre, X. , & Albuisson, J. (2017). Mutation spectrum in the ABCC6 gene and genotype‐phenotype correlations in a French cohort with pseudoxanthoma elasticum. Genetics in Medicine, 19(8), 909–917. 10.1038/gim.2016.213 28102862

[humu24477-bib-0029] Letavernier, E. , Kauffenstein, G. , Huguet, L. , Navasiolava, N. , Bouderlique, E. , Tang, E. , Delaitre, L. , Bazin, D. , de Frutos, M. , Gay, C. , Perez, J. , Verpont, M.‐C. , Haymann, J.‐P. , Pomozi, V. , Zoll, J. , Le Saux, O. , Daudon, M. , Leftheriotis, G. , & Martin, L. (2018). ABCC6 deficiency promotes development of randall plaque. Journal of the American Society of Nephrology, 29(9), 2337–2347. 10.1681/ASN.2017101148 29991491 PMC6115671

[humu24477-bib-0030] Levy‐Litan, V. , Hershkovitz, E. , Avizov, L. , Leventhal, N. , Bercovich, D. , Chalifa‐Caspi, V. , Manor, E. , Buriakovsky, S. , Hadad, Y. , Goding, J. , & Parvari, R. (2010). Autosomal‐recessive hypophosphatemic rickets is associated with an inactivation mutation in the ENPP1 gene. The American Journal of Human Genetics, 86(2), 273–278. 10.1016/j.ajhg.2010.01.010 20137772 PMC2820183

[humu24477-bib-0031] Li, Q. , Brodsky, J. L. , Conlin, L. K. , Pawel, B. , Glatz, A. C. , Gafni, R. I. , Schurgers, L. , Uitto, J. , Hakonarson, H. , Deardorff, M. A. , & Levine, M. A. (2014). Mutations in the ABCC6 gene as a cause of generalized arterial calcification of infancy: Genotypic overlap with pseudoxanthoma elasticum. Journal of Investigative Dermatology, 134(3), 658–665. 10.1038/jid.2013.370 24008425 PMC3945730

[humu24477-bib-0032] Li, Q. , Guo, H. , Chou, D. W. , Berndt, A. , Sundberg, J. P. , & Uitto, J. (2013). Mutant Enpp1asj mice as a model for generalized arterial calcification of infancy. Disease Models & Mechanisms, 6(5), 1227–1235. 10.1242/dmm.012765 23798568 PMC3759342

[humu24477-bib-0033] Li, Q. , Kingman, J. , Sundberg, J. P. , Levine, M. A. , & Uitto, J. (2016). Dual effects of bisphosphonates on ectopic skin and vascular soft tissue mineralization versus bone microarchitecture in a mouse model of generalized arterial calcification of infancy. Journal of Investigative Dermatology, 136(1), 275–283. 10.1038/JID.2015.377 26763447 PMC4731049

[humu24477-bib-0034] Lorenz‐Depiereux, B. , Schnabel, D. , Tiosano, D. , Häusler, G. , & Strom, T. M. (2010). Loss‐of‐function ENPP1 mutations cause both generalized arterial calcification of infancy and autosomal‐recessive hypophosphatemic rickets. The American Journal of Human Genetics, 86(2), 267–272. 10.1016/j.ajhg.2010.01.006 20137773 PMC2820166

[humu24477-bib-0035] Mackenzie, N. C. W. , Zhu, D. , Milne, E. M. , van 't Hof, R. , Martin, A. , Quarles, D. L. , Millán, J. L. , Farquharson, C. , & MacRae, V. E. (2012). Altered bone development and an increase in FGF‐23 expression in Enpp1(‐/‐) mice. PloS One, 7(2), e32177. 10.1371/journal.pone.0032177 22359666 PMC3281127

[humu24477-bib-0036] Maulding, N. D. , Kavanagh, D. , Zimmerman, K. , Coppola, G. , Carpenter, T. O. , Jue, N. K. , & Braddock, D. T. (2021). Genetic pathways disrupted by ENPP1 deficiency provide insight into mechanisms of osteoporosis, osteomalacia, and paradoxical mineralization. Bone, 142, 115656. 10.1016/j.bone.2020.115656 32980560 PMC7744330

[humu24477-bib-0037] Mulcahy, C. H. , Mone, F. , McAuliffe, F. M. , Mooney, E. , McParland, P. , & Mc Mahon, C. J. (2019). Antenatal diagnosis of idiopathic infantile arterial calcification (IIAC): A single centre experience and review of the literature. Journal of Congenital Cardiology, 3(1), 1. 10.1186/s40949-018-0022-1

[humu24477-bib-0038] Nakamura, I. , Ikegawa, S. , Nakamura, Y. , Okuda, S. , Koshizuka, Y. , Kawaguchi, H. , Nakamura, K. , Koyama, T. , Goto, S. , Toguchida, J. , Matsushita, M. , Ochi, T. , Takaoka, K. , & Nakamura, Y. (1999). Association of the human NPPS gene with ossification of the posterior longitudinal ligament of the spine (OPLL. Human Genetics, 104(6), 492–497. 10.1007/s004390050993 10453738

[humu24477-bib-0039] Nam, H. K. , Liu, J. , Li, Y. , Kragor, A. , & Hatch, N. E. (2011). Ectonucleotide pyrophosphatase/phosphodiesterase‐1 (ENPP1) protein regulates osteoblast differentiation. Journal of Biological Chemistry, 286(45), 39059–39071. 10.1074/jbc.M111.221689 21930712 PMC3234731

[humu24477-bib-0040] Nitschke, Y. , Baujat, G. , Botschen, U. , Wittkampf, T. , du Moulin, M. , Stella, J. , Le Merrer, M. , Guest, G. , Lambot, K. , Tazarourte‐Pinturier, M.‐F. , Chassaing, N. , Roche, O. , Feenstra, I. , Loechner, K. , Deshpande, C. , Garber, S. J. , Chikarmane, R. , Steinmann, B. , Shahinyan, T. , … Rutsch, F. (2012). Generalized arterial calcification of infancy and pseudoxanthoma elasticum can be caused by mutations in either ENPP1 or ABCC6. The American Journal of Human Genetics, 90(1), 25–39. 10.1016/j.ajhg.2011.11.020 22209248 PMC3257960

[humu24477-bib-0041] Nitschke, Y. , Yan, Y. , Buers, I. , Kintziger, K. , Askew, K. , & Rutsch, F. (2018). ENPP1‐Fc prevents neointima formation in generalized arterial calcification of infancy through the generation of AMP. Experimental & Molecular Medicine, 50(10), 1–12. 10.1038/s12276-018-0163-5 PMC6204430 30369595

[humu24477-bib-0042] Nykamp, K. , Anderson, M. , Powers, M. , Garcia, J. , Herrera, B. , Ho, Y.‐Y. , Kobayashi, Y. , Patil, N. , Thusberg, J. , Westbrook, M. , The Invitae Clinical Genomics Group, G. , & Topper, S. (2017). Sherloc: A comprehensive refinement of the ACMG‐AMP variant classification criteria. Genetics in Medicine, 19(10), 1105–1117. 10.1038/gim.2017.37 28492532 PMC5632818

[humu24477-bib-0043] Oheim, R. , Zimmerman, K. , Maulding, N. D. , Stürznickel, J. , von Kroge, S. , Kavanagh, D. , Stabach, P. R. , Kornak, U. , Tommasini, S. M. , Horowitz, M. C. , Amling, M. , Thompson, D. , Schinke, T. , Busse, B. , Carpenter, T. O. , & Braddock, D. T. (2020). Human heterozygous ENPP1 deficiency is associated with early onset osteoporosis, a phenotype recapitulated in a mouse model of Enpp1 deficiency. Journal of Bone and Mineral Research, 35(3), 528–539. 10.1002/jbmr.3911 31805212 PMC7184798

[humu24477-bib-0044] Okawa, A. , Nakamura, I. , Goto, S. , Moriya, H. , Nakamura, Y. , & Ikegawa, S. (1998). Mutation in Npps in a mouse model of ossification of the posterior longitudinal ligament of the spine. Nature Genetics, 19(3), 271–273. 10.1038/956 9662402

[humu24477-bib-0045] Patel, M. , Andronikou, S. , Solomon, R. , Sinclair, P. , & McCulloch, M. (2004). Idiopathic arterial calcification in childhood. Pediatric Radiology, 34(8), 652–655. 10.1007/s00247-004-1166-z 15029465

[humu24477-bib-0046] Pfendner, E. G. , Vanakker, O. M. , Terry, S. F. , Vourthis, S. , McAndrew, P. E. , McClain, M. R. , Fratta, S. , Marais, A.‐S. , Hariri, S. , Coucke, P. J. , Ramsay, M. , Viljoen, D. , Terry, P. F. , De Paepe, A. , Uitto, J. , & Bercovitch, L. G. (2007). Mutation detection in the ABCC6 gene and genotype phenotype analysis in a large international case series affected by pseudoxanthoma elasticum. Journal of Medical Genetics, 44(10), 621–628. 10.1136/jmg.2007.051094 17617515 PMC2597973

[humu24477-bib-0047] Pomozi, V. , Brampton, C. , Fülöp, K. , Chen, L.‐H. , Apana, A. , Li, Q. , Uitto, J. , Le Saux, O. , & Váradi, A. (2014). Analysis of pseudoxanthoma elasticum‐causing missense mutants of ABCC6 in vivo; pharmacological correction of the mislocalized proteins. Journal of Investigative Dermatology, 134(4), 946–953. 10.1038/jid.2013.482 24352041 PMC3962510

[humu24477-bib-0048] Pomozi, V. , Brampton, C. , Szeri, F. , Dedinszki, D. , Kozák, E. , van de Wetering, K. , Hopkins, H. , Martin, L. , Váradi, A. , & Le Saux, O. (2017). Functional rescue of ABCC6 deficiency by 4‐phenylbutyrate therapy reduces dystrophic calcification in Abcc6‐/‐ mice. Journal of Investigative Dermatology, 137(3), 595–602. 10.1016/j.jid.2016.10.035 27826008 PMC5326614

[humu24477-bib-0049] Pomozi, V. , Brampton, C. , van de Wetering, K. , Zoll, J. , Calio, B. , Pham, K. , Owens, J. B. , Marh, J. , Moisyadi, S. , Váradi, A. , Martin, L. , Bauer, C. , Erdmann, J. , Aherrahrou, Z. , & Le Saux, O. (2017). Pyrophosphate supplementation prevents chronic and acute calcification in ABCC6‐deficient mice. The American Journal of Pathology, 187(6), 1258–1272. 10.1016/j.ajpath.2017.02.009 28416300 PMC5455066

[humu24477-bib-0050] Ramsay, M. , Greenberg, T. , Lombard, Z. , Labrum, R. , Lubbe, S. , Aron, S. , Marais, A.‐S. , Terry, S. , Bercovitch, L. , & Viljoen, D. (2009). Spectrum of genetic variation at the ABCC6 locus in South Africans: Pseudoxanthoma elasticum patients and healthy individuals. Journal of Dermatological Science, 54(3), 198–204. 10.1016/j.jdermsci.2009.02.008 19339160

[humu24477-bib-0051] Ran, Y. , & Thibodeau, P. H. (2017). Stabilization of nucleotide binding domain dimers rescues ABCC6 mutants associated with Pseudoxanthoma Elasticum. Journal of Biological Chemistry, 292(5), 1559–1572. 10.1074/jbc.M116.759811 27994049 PMC5290935

[humu24477-bib-0052] Richards, S. , Aziz, N. , Bale, S. , Bick, D. , Das, S. , Gastier‐Foster, J. , Grody, W. W. , Hegde, M. , Lyon, E. , Spector, E. , Voelkerding, K. , & Rehm, H. L. , ACMG Laboratory Quality Assurance Committee . (2015). Standards and guidelines for the interpretation of sequence variants: A joint consensus recommendation of the American College of Medical Genetics and Genomics and the Association for Molecular Pathology. Genetics in Medicine, 17(5), 405–424. 10.1038/gim.2015.30 25741868 PMC4544753

[humu24477-bib-0053] Rutsch, F. , Böyer, P. , Nitschke, Y. , Ruf, N. , Lorenz‐Depierieux, B. , Wittkampf, T. , Weissen‐Plenz, G. , Fischer, R.‐J. , Mughal, Z. , Gregory, J. W. , Davies, J. H. , Loirat, C. , Strom, T. M. , Schnabel, D. , Nürnberg, P. , & Terkeltaub, R. , GACI Study Group . (2008). Hypophosphatemia, hyperphosphaturia, and bisphosphonate treatment are associated with survival beyond infancy in generalized arterial calcification of infancy. Circulation: Cardiovascular Genetics, 1(2), 133–140. 10.1161/CIRCGENETICS.108.797704 20016754 PMC2794045

[humu24477-bib-0054] Rutsch, F. , Ruf, N. , Vaingankar, S. , Toliat, M. R. , Suk, A. , Höhne, W. , Schauer, G. , Lehmann, M. , Roscioli, T. , Schnabel, D. , Epplen, J. T. , Knisely, A. , Superti‐Furga, A. , McGill, J. , Filippone, M. , Sinaiko, A. R. , Vallance, H. , Hinrichs, B. , Smith, W. , … Nürnberg, P. (2003). Mutations in ENPP1 are associated with “idiopathic” infantile arterial calcification. Nature Genetics, 34(4), 379–381. 10.1038/ng1221 12881724

[humu24477-bib-0055] Saito, T. , Shimizu, Y. , Hori, M. , Taguchi, M. , Igarashi, T. , Fukumoto, S. , & Fujitab, T. (2011). A patient with hypophosphatemic rickets and ossification of posterior longitudinal ligament caused by a novel homozygous mutation in ENPP1 gene. Bone, 49(4), 913–916. 10.1016/j.bone.2011.06.029 21745613

[humu24477-bib-0056] Le Saux, O. , Fülöp, K. , Yamaguchi, Y. , Iliás, A. , Szabó, Z. , Brampton, C. N. , Pomozi, V. , Huszár, K. , Arányi, T. , & Váradi, A. (2011). Expression and in vivo rescue of human ABCC6 disease‐causing mutants in mouse liver. PLoS One, 6(9), e24738. 10.1371/journal.pone.0024738 21935449 PMC3173462

[humu24477-bib-0057] Sim, N.‐L. , Kumar, P. , Hu, J. , Henikoff, S. , Schneider, G. , & Ng, P. C. (2012). SIFT web server: Predicting effects of amino acid substitutions on proteins. Nucleic Acids Research, 40(Web Server issue), W452–W457. 10.1093/nar/gks539 22689647 PMC3394338

[humu24477-bib-0058] Stella, J. , Buers, I. , van de Wetering, K. , Höhne, W. , Rutsch, F. , & Nitschke, Y. (2016). Effects of different variants in the ENPP1 gene on the functional properties of ectonucleotide pyrophosphatase/phosphodiesterase family member 1. Human Mutation, 37(11), 1190–1201. 10.1002/humu.23057 27467858

[humu24477-bib-0059] Swango, K. L. , Demirkol, M. , Hüner, G. , Pronicka, E. , Sykut‐Cegielska, J. , Schulze, A. , & Wolf, B. (1998). Partial biotinidase deficiency is usually due to the D444H mutation in the biotinidase gene. Human Genetics, 102(5), 571–575. 10.1007/s004390050742 9654207

[humu24477-bib-0060] Theng, E. H. , Brewer, C. C. , Oheim, R. , Zalewski, C. K. , King, K. A. , Delsmann, M. M. , Rolvien, T. , Gafni, R. I. , Braddock, D. T. , Jeffrey Kim, H. , & Ferreira, C. R. (2022). Characterization of hearing‐impairment in generalized arterial calcification of infancy (GACI. Orphanet Journal of Rare Diseases, 17(1), 273. 10.1186/s13023-022-02410-w 35854274 PMC9295326

[humu24477-bib-0061] Thumbigere‐Math, V. , Alqadi, A. , Chalmers, N. I. , Chavez, M. B. , Chu, E. Y. , Collins, M. T. , Ferreira, C. R. , FitzGerald, K. , Gafni, R. I. , Gahl, W. A. , Hsu, K. S. , Ramnitz, M. S. , Somerman, M. J. , Ziegler, S. G. , & Foster, B. L. (2018). Hypercementosis associated with ENPP1 mutations and GACI. Journal of Dental Research, 97(4), 432–441. 10.1177/0022034517744773 29244957 PMC5863873

[humu24477-bib-0062] Tian, C. , Harris, B. S. , & Johnson, K. R. (2016). Ectopic mineralization and conductive hearing loss in Enpp1asj mutant mice, a new model for otitis media and tympanosclerosis. PLoS One, 11(12), e0168159. 10.1371/journal.pone.0168159 27959908 PMC5154548

[humu24477-bib-0063] Vaingankar, S. M. , Fitzpatrick, T. A. , Johnson, K. , Goding, J. W. , Maurice, M. , & Terkeltaub, R. (2004). Subcellular targeting and function of osteoblast nucleotide pyrophosphatase phosphodiesterase 1. American Journal of Physiology‐Cell Physiology, 286(5), C1177–C1187. 10.1152/ajpcell.00320.2003 15075217

[humu24477-bib-0064] Zhou, X. , Cui, Y. , Zhou, X. , & Han, J. (2012). Phosphate/pyrophosphate and MV‐related proteins in mineralisation: Discoveries from mouse models. International Journal of Biological Sciences, 8(6), 778–790. 10.7150/ijbs.4538 22719218 PMC3372882

[humu24477-bib-0065] Ziegler, S. G. , Gahl, W. A. , & Ferreira, C. R. (1993). Generalized Arterial Calcification of Infancy. In (Eds.) M. P. Adam , H. H. Ardinger , R. A. Pagon , S. E. Wallace , L. J. Bean , K. W. Gripp , G. M. Mirzaa & A. Amemiya , GeneReviews®. University of Washington. http://www.ncbi.nlm.nih.gov/books/NBK253403/ 25392903

[humu24477-bib-0066] Zimmerman, K. , Liu, X. , von Kroge, S. , Stabach, P. , Lester, E. R. , Chu, E. Y. , Srivastava, S. , Somerman, M. J. , Tommasini, S. M. , Busse, B. , Schinke, T. , Carpenter, T. O. , Oheim, R. , & Braddock, D. T. (2022). Catalysis‐Independent enpp1 protein signaling regulates mammalian bone mass. Journal of Bone and Mineral Research: The Official Journal of the American Society for Bone and Mineral Research, 37, 1733–1749. 10.1002/jbmr.4640 35773783 PMC9709593

