# Supplementary material for: ENPP1 deficiency: A clinical update on the relevance of individual variants using a locus‐specific patient database
Source: Hum Mutat. 2022 Oct 8;43(12):1673–705. doi: 10.1002/humu.24477 (PMC12465099; doi:10.1002/humu.24477)
Supplement: Supplementary file 1 — Supplementary information. [file HUMU-43--s001.docx]

**Journal:** *Human Mutation*

**Type of submission:** Mutation Update

**Development of a Comprehensive, Locus-specific Patient Database for ENPP1 Deficiency**

**to Clarify the Clinical Relevance of Individual Variants**

**SUPPLEMENTAL MATERIALS**

## ***Supplemental Methods***

## **Overview of Data Sources and Curation Process**

Mastermind is a database of variants with evidence cited in the medical literature that is assembled by Genomenon, Inc. For every gene in the human genome, variant lists are produced by indexing the titles and abstracts of greater than 30 million articles, the full text including figures and tables of more than 7 million articles prioritized to have genetic and genomic content, and more than 2 million genomic supplemental datasets. Each data source is initially indexed using a search for all synonyms of every gene in the human genome. Once a gene symbol or synonym is identified in the text of any of these references, the rest of the text is indexed using proprietary Genomic Language Processing to identify genetic variants. This process recognizes many different variant descriptions including cDNA, protein and rsID nomenclatures across all possible transcripts for each gene, including legacy and colloquial variant nomenclatures as well.

For *ENPP1* we followed stated guidelines for performing meta-analyses from biomedical literature (Forero et al., 2019). The goal of our work was to identify all genetic variants by curating all relevant references in the biomedical literature as identified using Mastermind and interpreting the clinical significance according to the clinically accepted literature curation and variant interpretation framework promulgated by the American College of Medical Genetics and Genomics/Association for Molecular Pathology (ACMG/AMP) (Richards et al., 2015). Case studies, case series, functional studies and review articles were considered for this meta-analysis. The details of the evidence being adjudicated is presented below in greater detail. Included also in the variant interpretation process was consideration of population frequency of each variant and in silico predictions of likely damaging effects to protein function. Prior to completion of the curation, available data from other third-party databases including ClinVar and other locus-specific databases that have identified additional pathogenic or likely pathogenic variants was consulted to ensure maximal sensitivity of the resulting dataset. Wherever possible, an effort was made to contact the original authors of references for clarification if needed. A complete accounting of the results of the literature curation process and data used for the ACMG interpretation process are provided.

##

## **Variant Mapping to Chromosomal Coordinates**

Variants found in the literature are first associated with their genomic level change. In Mastermind, the process of mapping variants in any gene to chromosomal coordinates begins by gathering textual references for each variant from the literature as described above. For example, the *BRCA1* D693N variant includes the following textual references, among many other possible nomenclatures: p.Asp693Asn, c.2077G>A, rs4986850, D693N, and c.2077G > A. A set of possible Human Genetic Variation Society (HGVS) protein and cDNA effects is then constructed from these textual references by finding the RefSeq protein or transcript sequences for which the given effect is possible. Continuing with the *BRCA1* D693N example, the following HGVS protein effects can be inferred from the text: NP_009231.2:p.Asp693Asn and NP_009225.1:p.Asp693Asn. And the following HGVS cDNA effects can be inferred: *NM_007300.3*:c.2077G>A and *NM_007294.3*:c.2077G>A. For rsID references, the set of possible protein and cDNA effects are cross-referenced using dbSNP build151. Additional HGVS cDNA effects are then generated by expanding the HGVS representation for any single amino acid protein substitution into the set of possible HGVS cDNA variants that could produce the given amino acid effect. In our *BRCA1* D693N example above the following cDNA effects would be added by this process: *NM_007294.3*:c.2077_2079delGACinsAAT and *NM_007300.3*:c.2077_2079delGACinsAAT.

**Evidence from Population Frequency Databases**

All GRCh37 Single Nucleotide Variants (SNVs) found, with a single-nucleotide ref or single-nucleotide alt allele, are cross-referenced with the gnomAD v2.1.1 VCF files[^[3]^](https://usc-word-edit.officeapps.live.com/we/wordeditorframe.aspx?ui=en%2DUS&rs=en%2DUS&wopisrc=https%3A%2F%2Fgenomenon.sharepoint.com%2F_vti_bin%2Fwopi.ashx%2Ffiles%2Fd51b263cc9d649638694a2d6a3f2943f&wdenableroaming=1&mscc=1&hid=CF89DB9F-9015-C000-51CF-E1F79F79518E&wdorigin=Other&jsapi=1&jsapiver=v1&newsession=1&corrid=c27c01ee-222b-30d0-57f9-315cb8b28a8b&usid=c27c01ee-222b-30d0-57f9-315cb8b28a8b&sftc=1&mtf=1&sfp=1&instantedit=1&wopicomplete=1&wdredirectionreason=Unified_SingleFlush&preseededsessionkey=05eb3b64-f7d7-d1b0-7bfa-745917deab15&preseededwacsessionid=c27c01ee-222b-30d0-57f9-315cb8b28a8b&rct=Medium&ctp=LeastProtected#_ftn3). All GRCh37 Multi-nucleotide Variants (MNVs), with 2 nucleotides in each of the ref and alt alleles, are cross-referenced in the gnomAD v2.1.1 Coding MNVs dataset published at the same location.

For any coding variants that are not amino acid substitutions and do not represent a gained or lost stop codon, the gnomAD data is referenced using the Variant Effect Predictor (VEP) annotations provided in the gnomAD VCF records. Any gnomAD records with VEP protein effect predictions that match the protein position, reference amino acid, and effect type (frameshift, deletion, insertion, duplication, or inversion) of the variant are included in the results for the variant, regardless of alternate amino acid(s) or length. In addition, the GRCh37 (hg19) genomic variant mappings included in the gnomAD records matched in this way are added to the set of chromosomal variants found in the “Mapping to Chromosomal Coordinates” process above.

If the total minor allele frequency or any individual population minor allele frequency for any chromosomal variant that results in the protein or cDNA effect in question is greater than 0.05 in the gnomAD dataset, the given protein or cDNA effect is considered to be “common” for the purposes of ACMG classification.

Likewise, if no chromosomal variants for a given protein or cDNA effect are found to be “common”, and if the minor allele frequency is missing from the gnomAD dataset or less than 0.005 or *all* populations and for *all* chromosomal variants for a given protein or cDNA effect, the given protein or cDNA effect is considered to be “rare” for the purposes of ACMG classification.

##

## **Evidence from Damage Prediction Algorithms**

GRCh37 SNVs derived from the process above are cross-referenced against the dbNSFP v4.0 database[^[4]^](https://usc-word-edit.officeapps.live.com/we/wordeditorframe.aspx?ui=en%2DUS&rs=en%2DUS&wopisrc=https%3A%2F%2Fgenomenon.sharepoint.com%2F_vti_bin%2Fwopi.ashx%2Ffiles%2Fd51b263cc9d649638694a2d6a3f2943f&wdenableroaming=1&mscc=1&hid=CF89DB9F-9015-C000-51CF-E1F79F79518E&wdorigin=Other&jsapi=1&jsapiver=v1&newsession=1&corrid=c27c01ee-222b-30d0-57f9-315cb8b28a8b&usid=c27c01ee-222b-30d0-57f9-315cb8b28a8b&sftc=1&mtf=1&sfp=1&instantedit=1&wopicomplete=1&wdredirectionreason=Unified_SingleFlush&preseededsessionkey=05eb3b64-f7d7-d1b0-7bfa-745917deab15&preseededwacsessionid=c27c01ee-222b-30d0-57f9-315cb8b28a8b&rct=Medium&ctp=LeastProtected#_ftn4). Any SIFT, SIFT4G, PolyPhen-2 HVAR, or PolyPhen-2 HDIV damage predictions from that dataset that match the protein effect of the Mastermind Reporter variant being annotated will be used to inform ACMG calls for the variant.

If any chromosomal variants resulting in the protein or cDNA effect in question are classified as “damaging” in SIFT or SIFT4G and PolyPhen-2 HDIV or HVAR, the protein or cDNA effect in question is categorized as “damaging” for the purposes of the ACMG classification. If no chromosomal variants associated with the effect and classified as “damaging” in SIFT or SIFT4G and PolyPhen-2 HDIV or HVAR and at least one chromosomal variant is classified as “tolerated” or “benign” in SIFT or SIFT4G and PolyPhen-2 HDIV or HVAR, the protein or cDNA effect in question is categorized as “tolerated”.

# Evidence from Literature Curations and Variant Interpretation Evidence Categories

Our literature curation and variant interpretation process follows the industry standard guidelines as closely as possible. In the case of constitutional disease, this interpretation framework is discussed in detail in Richards *et al.* 2015 (Richards et al., 2015). Each variant cited in each of the references is examined and manually scored according to this framework. Specific details and curation parameters for each category of evidence are provided below.

###

### **Pathogenic Very Strong Evidence Category**

PVS1 – *Loss of function variants in a gene where loss of function is a known pathogenic mechanism.* This category applies to all frameshift and nonsense variants with a loss of function mechanism of pathogenicity. Consideration is given to documented splice site variants if functional evidence is provided by authors illustrating a loss of function at the protein level. For gain of function mechanisms of disease development, this category of evidence is not considered. In the case of frameshift and nonsense variants, the use of this category of evidence is intrinsic to the variant change itself and no insight from the literature or third-party database is needed. Proximity of the variant to the 3’ end of the transcript is taken into account and noted where potentially relevant to an absence of potential consequences to protein function.

###

### **Pathogenic Strong Evidence Categories**

PS1 – *Different nucleotide change as a previously established pathogenic variant with the same protein consequence.* For these types of variants, the data from Mastermind includes these variants together.

PS2 – *De novo inheritance with paternity and maternity confirmed in a patient with disease and no family history.* Where identified in the literature, this category of evidence is noted.

PS3 – *Well-established in vitro or in vivo functional studies supportive of a damaging effect on the gene or gene product.* All functional studies demonstrating a functional consequence of the genetic variant on protein function are annotated with the assay and the functional consequence.

PS4 – *The prevalence of the variant in affected individuals is significantly increased compared to the prevalence in controls.* This information comes from review of literature containing cohort studies including at least two unrelated affected individuals.

###

### **Pathogenic Moderate Evidence Categories**

PM1 – *Variant in hotspot or functional domain without frequent benign variation*. If a known hotspot region is identified, variants within this region are annotated with this category of evidence provided the PS1 evidence category has not already been used.

PM2 – *Absent from large population studies or at extremely low frequency when recessive.* Details of how variant population frequency is determined is provided above in the Evidence from Population Frequency Databases section.

PM3 – *For recessive disorders, variant detected in trans with a pathogenic variant.* Where identified in the literature, this category of evidence is noted.

PM4 – *Protein-length changes due to in-frame deletions/insertions in a non-repeat region or stop-loss variant.* Where identified in the literature, this category of evidence is noted.

PM5 – *Missense change at an amino acid residue where a different missense change previously established as pathogenic.* Where appropriate after surveying the list of pathogenic and likely pathogenic variants, this category of evidence is noted.

PM6 – *Assumed de novo without confirmation of paternity and maternity.* This evidence category requires a clinical laboratory result and is not usually found in literature. If found in literature, it is used instead of PS2 if paternity and maternity are not listed as having been confirmed.

### **Pathogenic Supporting Evidence Categories**

PPC – *An isolated case report demonstrating the variant in an affected individual*. This criterion is an addition to the ACMG/AMP framework having been added to capture evidence that in aggregate indicates a pathogenic association between the variant and disease.

PP1 – *Variant segregates with disease in multiple affected family members*. Where identified in the literature, this category of evidence is noted and distinguished from PS4 above by the presence of a family as opposed to unrelated individuals.

PP2 – *Missense variant in a gene that has a low rate of benign missense variation and where missense variants are a common mechanism of disease.* Determination of missense being a typical mode of pathogenesis for a given gene as well as the assessment of a low rate of benign variation in the gene is determined using aggregate information from gnomAD using the pLoF (pLI) score or the ratio of expected to observed genetics variations as a guide (Karczewski et al., 2020).

PP3 – *Multiple lines of computational evidence support a deleterious effect on the gene or gene product.* Details of how predicted deleterious effect is determined is provided above in the Evidence from Damage Prediction Algorithms section. Occasionally, insight from the literature will describe results from *in silico* prediction models of pathogenicity. These are included in the annotations but are not used toward final pathogenicity calculations.

PP4 – *Patient’s phenotype or family history is highly specific for a disease with a single genetic etiology.* This is a clinical determination and is not relevant to literature curation or database assembly.

###

### **Benign Stand-Alone Evidence Category**

BA1 – *Allele frequency is 5%*. This information is extracted from the gnomAD database as detailed above in the Evidence from Population Frequency Databases section.

###

### **Benign Strong Evidence Category**

BS1 – *Allele frequency is too high for disorder prevalence*. This evidence is invoked if the allele frequency is higher than a baseline allele frequency of 1% but can be adjusted as needed if proper disease prevalence information is available.

BS2 – *Observed in a healthy adult individual for a recessive, dominant or X-linked disorder with full penetrance expected at an early age*. Where identified in the literature, this category of evidence is noted.

BS3 – *Functional studies of mammalian knock-in models supportive of no damaging effect on protein function or splicing.* All functional studies demonstrating a lack of functional consequence of the genetic variant on protein function are annotated with the assay and the functional consequence being assayed.

BS4 – *Lack of segregation in affected members of a family*. Where identified in the literature, this category of evidence is noted.

### **Benign Supporting Evidence Category**

BP1 – *Missense variant in a gene for which only truncating variants are known to be pathogenic*. Where identified in the literature, this category of evidence is noted.

BP2 – *Observed in trans with a pathogenic variant for a fully penetrant dominant gene/disorder or observed in cis with a pathogenic variant in any inheritance pattern*. Where identified in the literature, this category of evidence is noted.

BP3 – *In-frame deletions or insertions in a repetitive region without a known function*. Where identified in the literature, this category of evidence is noted.

BP4 – *Multiple lines of computational evidence suggest no impact on gene or gene product*.

BP5 – *Variant found in a case with an alternate molecular basis for disease*. Where identified in the literature, this category of evidence is noted.

BP6 – *Reputable source recently reports the variant as benign*. This criterion is not utilized as it is a highly controversial inclusion in the ACMG/AMP framework.

BP7 – *A silent variant for which splicing prediction algorithms predict no impact to the splice consensus site nor creation of a new splice site*. If splicing effects are suggested, this category is not included.

**SUPPLEMENTAL REFERENCES**

Forero DA, Lopez-Leon S, González-Giraldo Y, Bagos PG. Ten simple rules for carrying out and writing meta-analyses. PLoS Comput Biol. 2019 May 16;15(5):e1006922. doi: 10.1371/journal.pcbi.1006922. PMID: 31095553; PMCID: PMC6521986.

Richards S, Aziz N, Bale S, Bick D, Das S, Gastier-Foster J, Grody WW, Hegde M, Lyon E, Spector E, Voelkerding K, Rehm HL; ACMG Laboratory Quality Assurance Committee. Standards and guidelines for the interpretation of sequence variants: a joint consensus recommendation of the American College of Medical Genetics and Genomics and the Association for Molecular Pathology. Genet Med. 2015 May;17(5):405-24. doi: 10.1038/gim.2015.30. Epub 2015 Mar 5. PMID: 25741868; PMCID: PMC4544753.

[^[3]^](https://usc-word-edit.officeapps.live.com/we/wordeditorframe.aspx?ui=en%2DUS&rs=en%2DUS&wopisrc=https%3A%2F%2Fgenomenon.sharepoint.com%2F_vti_bin%2Fwopi.ashx%2Ffiles%2Fd51b263cc9d649638694a2d6a3f2943f&wdenableroaming=1&mscc=1&hid=CF89DB9F-9015-C000-51CF-E1F79F79518E&wdorigin=Other&jsapi=1&jsapiver=v1&newsession=1&corrid=c27c01ee-222b-30d0-57f9-315cb8b28a8b&usid=c27c01ee-222b-30d0-57f9-315cb8b28a8b&sftc=1&mtf=1&sfp=1&instantedit=1&wopicomplete=1&wdredirectionreason=Unified_SingleFlush&preseededsessionkey=05eb3b64-f7d7-d1b0-7bfa-745917deab15&preseededwacsessionid=c27c01ee-222b-30d0-57f9-315cb8b28a8b&rct=Medium&ctp=LeastProtected#_ftnref3) <https://gnomad.broadinstitute.org/downloads>

[^[4]^](https://usc-word-edit.officeapps.live.com/we/wordeditorframe.aspx?ui=en%2DUS&rs=en%2DUS&wopisrc=https%3A%2F%2Fgenomenon.sharepoint.com%2F_vti_bin%2Fwopi.ashx%2Ffiles%2Fd51b263cc9d649638694a2d6a3f2943f&wdenableroaming=1&mscc=1&hid=CF89DB9F-9015-C000-51CF-E1F79F79518E&wdorigin=Other&jsapi=1&jsapiver=v1&newsession=1&corrid=c27c01ee-222b-30d0-57f9-315cb8b28a8b&usid=c27c01ee-222b-30d0-57f9-315cb8b28a8b&sftc=1&mtf=1&sfp=1&instantedit=1&wopicomplete=1&wdredirectionreason=Unified_SingleFlush&preseededsessionkey=05eb3b64-f7d7-d1b0-7bfa-745917deab15&preseededwacsessionid=c27c01ee-222b-30d0-57f9-315cb8b28a8b&rct=Medium&ctp=LeastProtected#_ftnref4) <https://sites.google.com/site/jpopgen/dbNSFP>

Karczewski KJ, Francioli LC, Tiao G, Cummings BB, Alföldi J, Wang Q, Collins RL, Laricchia KM, Ganna A, Birnbaum DP, Gauthier LD, Brand H, Solomonson M, Watts NA, Rhodes D, Singer-Berk M, England EM, Seaby EG, Kosmicki JA, Walters RK, Tashman K, Farjoun Y, Banks E, Poterba T, Wang A, Seed C, Whiffin N, Chong JX, Samocha KE, Pierce-Hoffman E, Zappala Z, O'Donnell-Luria AH, Minikel EV, Weisburd B, Lek M, Ware JS, Vittal C, Armean IM, Bergelson L, Cibulskis K, Connolly KM, Covarrubias M, Donnelly S, Ferriera S, Gabriel S, Gentry J, Gupta N, Jeandet T, Kaplan D, Llanwarne C, Munshi R, Novod S, Petrillo N, Roazen D, Ruano-Rubio V, Saltzman A, Schleicher M, Soto J, Tibbetts K, Tolonen C, Wade G, Talkowski ME; Genome Aggregation Database Consortium, Neale BM, Daly MJ, MacArthur DG. The mutational constraint spectrum quantified from variation in 141,456 humans. Nature. 2020 May;581(7809):434-443. doi: 10.1038/s41586-020-2308-7. Epub 2020 May 27. Erratum in: Nature. 2021 Feb;590(7846):E53. PMID: 32461654; PMCID: PMC7334197.

^[7]^ <https://www.nlm.nih.gov/mesh/meshhome.html>
